# c-MYC-directed NRF2 drives malignant progression of head and neck cancer via glucose-6-phosphate dehydrogenase and transketolase activation

**DOI:** 10.7150/thno.53417

**Published:** 2021-03-11

**Authors:** Ya-Chu Tang, Jenn-Ren Hsiao, Shih-Sheng Jiang, Jang-Yang Chang, Pei-Yi Chu, Ko-Jiunn Liu, Hsun-Lang Fang, Li-Mei Lin, Huang-Hui Chen, Yen-Wen Huang, Yu-Tsen Chen, Fang-Yu Tsai, Su-Fang Lin, Yung-Jen Chuang, Ching-Chuan Kuo

**Affiliations:** 1 Graduate Program of Medical Biotechnology, National Tsing Hua University, Hsinchu, Taiwan.; 2 Institute of Biotechnology and Pharmaceutical Research, National Health Research Institutes, Miaoli, Taiwan.; 3 Department of Otolaryngology, National Cheng Kung University Hospital, College of Medicine, National Cheng Kung University, Tainan, Taiwan.; 4 Institute of Clinical Medicine, College of Medicine, National Cheng Kung University, Tainan, Taiwan.; 5 National Institute of Cancer Research, National Health Research Institutes, Miaoli, Taiwan.; 6 Department of Internal Medicine, College of Medicine, National Cheng Kung University, Tainan, Taiwan.; 7 Department of Pathology, Show Chwan Memorial Hospital, Changhua, Taiwan.; 8 Department of Cosmetology and Health Care, Min-Hwei College of Health Care Management, Tainan, Taiwan.; 9 Institute of Bioinformatics and Structural Biology, National Tsing Hua University, Hsinchu, Taiwan.; 10 Department of Medical Science, National Tsing Hua University, Hsinchu, Taiwan.; 11 Graduate Institute of Biomedical Sciences, China Medical University, Taichung, Taiwan.

**Keywords:** Nuclear factor erythroid 2-related factor 2 (NRF2), head and neck squamous cell carcinoma (HNSCC), c-MYC, pentose phosphate pathway (PPP), glucose-6-phosphate dehydrogenase (G6PD), transketolase (TKT)

## Abstract

**Rationale:** NRF2, a redox sensitive transcription factor, is up-regulated in head and neck squamous cell carcinoma (HNSCC), however, the associated impact and regulatory mechanisms remain unclear.

**Methods:** The protein expression of NRF2 in HNSCC specimens was examined by IHC. The regulatory effect of c-MYC on NRF2 was validated by ChIP-qPCR, RT-qPCR and western blot. The impacts of NRF2 on malignant progression of HNSCC were determined through genetic manipulation and pharmacological inhibition *in vitro* and *in vivo*. The gene-set enrichment analysis (GSEA) on expression data of cDNA microarray combined with ChIP-qPCR, RT-qPCR, western blot, transwell migration/ invasion, cell proliferation and soft agar colony formation assays were used to investigate the regulatory mechanisms of NRF2.

**Results:** NRF2 expression is positively correlated with malignant features of HNSCC. In addition, carcinogens, such as nicotine and arecoline, trigger c-MYC-directed NRF2 activation in HNSCC cells. NRF2 reprograms a wide range of cancer metabolic pathways and the most notable is the pentose phosphate pathway (PPP). Furthermore, glucose-6-phosphate dehydrogenase (G6PD) and transketolase (TKT) are critical downstream effectors of NRF2 that drive malignant progression of HNSCC; the coherently expressed signature NRF2/G6PD/TKT gene set is a potential prognostic biomarker for prediction of patient overall survival. Notably, G6PD- and TKT-regulated nucleotide biosynthesis is more important than redox regulation in determining malignant progression of HNSCC.

**Conclusions:** Carcinogens trigger c-MYC-directed NRF2 activation. Over-activation of NRF2 promotes malignant progression of HNSCC through reprogramming G6PD- and TKT-mediated nucleotide biosynthesis. Targeting NRF2-directed cellular metabolism is an effective strategy for development of novel treatments for head and neck cancer.

## Introduction

Nuclear factor erythroid 2-related factor 2 (NRF2) is a member of Cap'n'Collar family of transcription factors that share a highly conserved basic region‐leucine zipper structure. NRF2 is tightly regulated by Kelch‐like ECH associating protein 1 (KEAP1), a substrate adaptor protein for a Cullin3‐based E3 ubiquitin ligase, resulting in expression of NRF2 at low transactivating levels in all human organs 1, 2. When cells are exposed to electrophiles or oxidative stress, NRF2 is released from KEAP1 and transported into the cell nucleus to activate the transcription of specific cytoprotective genes by binding to the antioxidant response element (ARE) as a heterodimer with the small Maf protein 1, 2. Abundant evidence has shown that activation of NRF2 can suppress carcinogenesis 3, 4. However, recent evidence has revealed that aberrant activation of NRF2 is associated with cancer hallmarks 4-10, including promoted tumorigenesis 11, 12, sustained proliferative signaling and survival advantage 7, 13, 14, resistance to treatment 15-18, deregulated redox homeostasis 19-21, enhanced tissue invasion and metastasis 14, 22, 23, and altered cellular metabolism 13, 24-26.

Head and neck squamous cell carcinoma (HNSCC) is the fifth most common malignancy worldwide and comprises a diverse set of cancers arising from the squamous epithelium of the oral cavity, larynx, and pharynx, including the nasopharynx, oropharynx, hypopharynx, etc. 27. The primary risk factors for HNSCC include alcohol consumption, betel nut chewing, cigarette smoking, human papillomavirus (HPV) infection (for oropharyngeal cancer), and Epstein-Barr virus (EBV) infection (for nasopharyngeal cancer) 28. Even though the curability of early-stage HNSCC is high, the survival rate of patients with recurrent or metastatic HNSCC diminishes drastically 29. Exploring robust biomarkers that contribute to the malignant progression of HNSCC may aid the development of useful therapeutic strategies. For example, identification of aberrant signaling pathways and targeting these molecules (e.g., EGFR) has been an effective approach to HNSCC treatment 30.

Genomic studies using The Cancer Genome Atlas Network revealed that the activation of the NRF2/KEAP1/CUL3 pathway is a common feature of HPV-negative HNSCC 29. In addition, several groups demonstrated that increased NRF2 expression was observed in HNSCC and positively correlated with poor clinical outcome 19, 31-33. There is limited research on the effects of NRF2 on the malignant progression of HNSCC, which includes NRF2-mediated radiation tolerance and drug resistance 16, 34, 35, activated oncogenic Notch 14 or mTOR signaling 36, and NRF2 activity of as a central node in the maintenance of low ROS levels and stemness properties 19. Thus, the biological consequences of NRF2 in head and neck cancer remain unclear, and the critical NRF2 downstream effectors that participate in NRF2-promoted malignant progression have yet to be conclusively identified in HNSCC. In this study, we provide evidence that activation of NRF2 induces tumorigenesis and promotes tumor growth and metastasis. We also report our discovery of potential upstream modulators and downstream effectors of NRF2 in head and neck cancer. Among them, we identified c-MYC, which is associated with chemical induced carcinogenesis and a modulator of NRF2 activation. In addition, glucose-6-phosphate dehydrogenase (G6PD) and transketolase (TKT) exhibit exclusive roles in determining how NRF2 drives malignant progression of HNSCC.

## Materials and Methods

### HNSCC patient samples

Human HNSCC clinical specimens at different clinicopathological stages were obtained from National Cheng Kung University Hospital, Tainan, Taiwan in accordance with the associated Institutional Review Board (A-ER-107-147) and National Health Research Institutes, Zhunan, Miaoli, Taiwan (EC1070105).

### Cell lines

Normal human oral keratinocytes (NHOK), human dysplastic oral keratinocytes (DOK), and human HNSCC cells (Ca9-22, HSC-3, OC3, HONE-1 and OEC-M1) were maintained as described previously 37-40. The Ca9-22-D1 cell line and the carcinogen-transformed DOK cells were established in Dr. Ching-Chuan Kuo's laboratory (**Figure S1**, **Figure S2**). See ***Supplementary Materials and Methods*** for details. All cell lines were authenticated using Short-tandem-repeat (STR) profiling (DNA fingerprinting).

### Genetic manipulation

For transient gene silencing, the siRNAs specific to NRF2, G6PD, and TKT and siRNA negative controls were purchased from Invitrogen and delivered into cells using Lipofectamine RNAiMAX reagent (Thermo Fisher Scientific, Waltham, MA., USA). For establishment of stable NRF2-knockdown HNSCC cells, a set of pGFP-C-shLenti vectors containing four independent 29-mer shRNAs against human NRF2 and a non-targeting shRNA were purchased from OriGene Technologies Inc. (Rockville, MD., USA). After transfection, the cells were selected by puromycin treatment. For generation of stable NRF2-overexpressing DOK cells, human NRF2 was amplified from Gene Pool^TM^ normal human prostate cDNA (Life Technologies, Carlsbad, CA., USA) and cloned into the pIRES2-EGFP vector between the Nhe1 and Xho1 cloning sites. The specific primers used for plasmid cloning are listed in **Table S1**. After transfection, the cells were selected by geneticin (G418) treatment.

### Evaluation of mRNA and protein levels

The real-time polymerase chain reaction (PCR) primer pairs used for amplification of target genes are listed in **Table S2**. Gene expression was determined by quantitative real-time PCR. Western blot analysis and immunohistochemistry (IHC) staining were used to analyze protein levels of cell lysates and tissue sections, respectively. The antibodies used for recognizing specific proteins are listed in **Table S3**.

### Assessment of malignant features

(1) *In vitro*: Cell proliferation was assessed by methylene blue staining. Anchorage-independent growth was examined using the soft agar colony formation assay. Trans-well migration and invasion assays were performed to evaluate cell motility and invasiveness, respectively. (2) *In vivo*: Animals used in this study were purchased from BioLASCO Taiwan Co., Ltd (Taipei, Taiwan) and maintained at the Laboratory Animal Center of National Health Research Institutes (NHRI), Taiwan. The *in vivo* tumorigenic assay and the experimental lung metastasis assay were performed. See ***Supplementary Materials and Methods*** for details.

### Chromatin immunoprecipitation-quantitative polymerase chain reaction (ChIP-qPCR)

Chromatin immunoprecipitation (ChIP) was performed using the Magna ChIP™ A/G Chromatin Immunoprecipitation Kit (Merck Millipore, Burlington, MA, USA) with an antibody specific for NRF2 (Abcam, Cambridge, MA., USA), c-MYC (Abcam, Cambridge, MA., USA) or normal rabbit IgG (Santa Cruz Biotechnology, Santa Cruz, CA., USA). Following ChIP, quantitative PCR was utilized to amplify and quantify the immunoprecipitated DNA using primers specific for the NRF2-targeted antioxidant response element (ARE) within G6PD or TKT, as well as primers for a c-MYC binding site within the NRF2 promoter region (**Table S4**). The c-MYC binding site within the NRF2 promoter region was obtained from the ENCODE Consortium. The ChIP-qPCR values were normalized to that of input control and represented as fold enrichment relative to the anti-normal rabbit IgG control.

### Statistical analysis

The experimental results were analyzed and expressed as the mean ± standard deviation (S.D.) or mean ± standard error (S.E.) for the *in vitro* and *in vivo* experiments, respectively. One-way analysis of variance (ANOVA) and Student's t-test were used to determine statistical significance.

## Results

### Expression of NRF2 is positively correlated with the malignant characteristics of HNSCC

**Figure 1:**
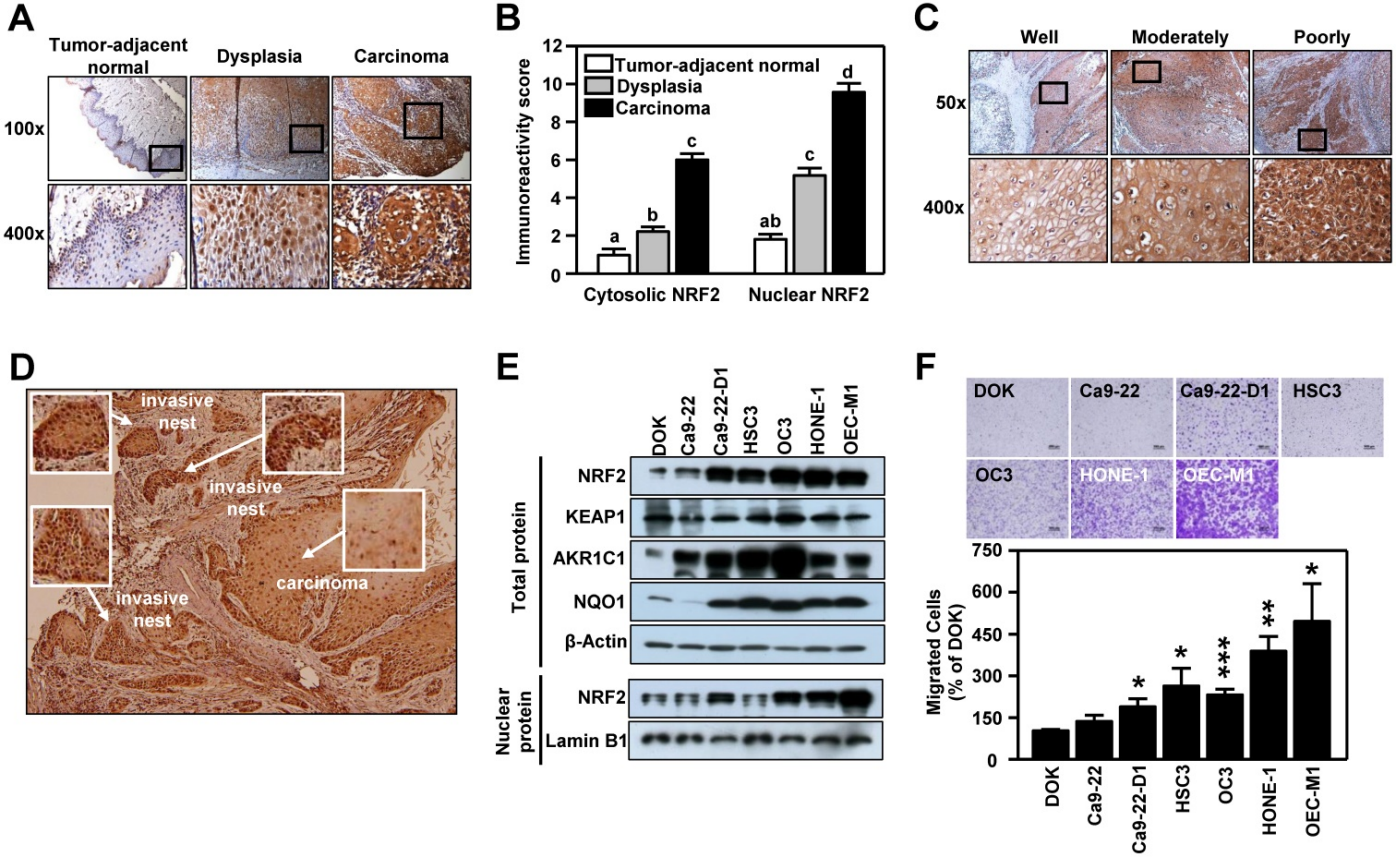
** NRF2 expression is positively associated with malignancy in HNSCC.** (A, C, D) Representative IHC staining of NRF2 in the specimens obtained from HNSCC patients. Brown staining indicates NRF2 positivity. The cell nuclei and background were counterstained with hematoxylin (blue coloration). (A) NRF2 protein expression in HNSCC specimens with different clinicopathological stages. Magnification, 100× (upper panel) and 400× (lower panel). (B) The correlation between the level of nuclear and cytosolic expression of NRF2 and tumor stage was estimated using the immunoreactivity scoring system (IRS). The IRS was calculated by multiplying the intensity of NRF2 staining (scale, 0-3) by the percentage of positive cells (4, > 80%; 3, 51-80%; 2, 10-50%; 1, < 10%; 0, 0%), which resulted in values ranging from 0 to 12. Groups with *different letters* are significant different (*p* < 0.05) from each other. (C) NRF2 protein expression was positively correlated with the degree of tumor cell differentiation in human head and neck squamous cell carcinoma. Magnification, 50× (upper panel) and 400× (lower panel). (D) NRF2 expression was highest in the invasive carcinoma nests. Magnification 50×. (E) Levels of NRF2 and NRF2 downstream targets in dysplasia oral keratinocyte (DOK) and HNSCC cell lines. (F) Motility of DOK and various HNSCC cell lines via trans-well migration assay. * *p* < 0.05; ** *p* < 0.01; *** *p* < 0.001.

The clinicopathological significance of NRF2 was validated in an in-house cohort of clinical specimens of HNSCC (n=77) by IHC staining. In tumor-adjacent normal epithelium, few cells with positive staining for NRF2 were detected and most of the NRF2-positive cells were found in the active para-basal and basal layers. Conversely, in the oral epithelial dysplastic lesions and oral squamous cell carcinoma, NRF2-positive cells spread into the intermediate layer and the staining intensity was dramatically higher than in cancer-adjacent normal epithelia (**Figure 1A**). Based on immuno-reactive score (IRS) analysis, we noted that NRF2 was localized predominantly in cell nuclei, and both nuclear and cytosolic NRF2 levels increased significantly with malignant progression, i.e., progression from tumor-adjacent normal epithelia to epithelial dysplasia to squamous cell carcinoma (**Figure 1B**). We also compared differential expression of *NFE2L2*, the gene encoding NRF2, in HNSCC and normal tissue from clinical cohorts using the online cancer microarray database Oncomine 41. As shown in **Table S5**, increased NRF2 expression in tumors compared to normal tissue appeared in many HNSCC datasets. While analyzing NRF2 expression with histological differentiation, we demonstrated that NRF2 level was positively correlated with the degree of differentiation (**Figure 1C**). Intriguingly, IHC analysis demonstrated higher staining intensity of NRF2 in the invasive oral squamous cell carcinoma and invasive nests than in primary carcinoma (**Figure 1D**). Similar to clinical findings, the expression of NRF2 protein was markedly higher in the HNSCC cell lines than in dysplasia oral keratinocytes (DOK; **Figure 1E**), and lower levels of NRF2 protein were observed in normal human oral keratinocytes (NHOK) compared to DOK and HNSCC cells (**Figure S3**). Increased nuclear localization of NRF2 led to upregulation of NRF2 downstream targets, such as AKR1C1, NQO1 (**Figure 1E**), and TrxR **(Figure S3)**. Notably, overexpression of NRF2 was positively related to motility of HNSCC cells (**Figure 1F**). These results indicate that expression of NRF2 is positively correlated with malignant features of HNSCC.

### NRF2 is upregulated by c-MYC and acts as an important determinant for malignant progression of HNSCC

**Figure 2:**
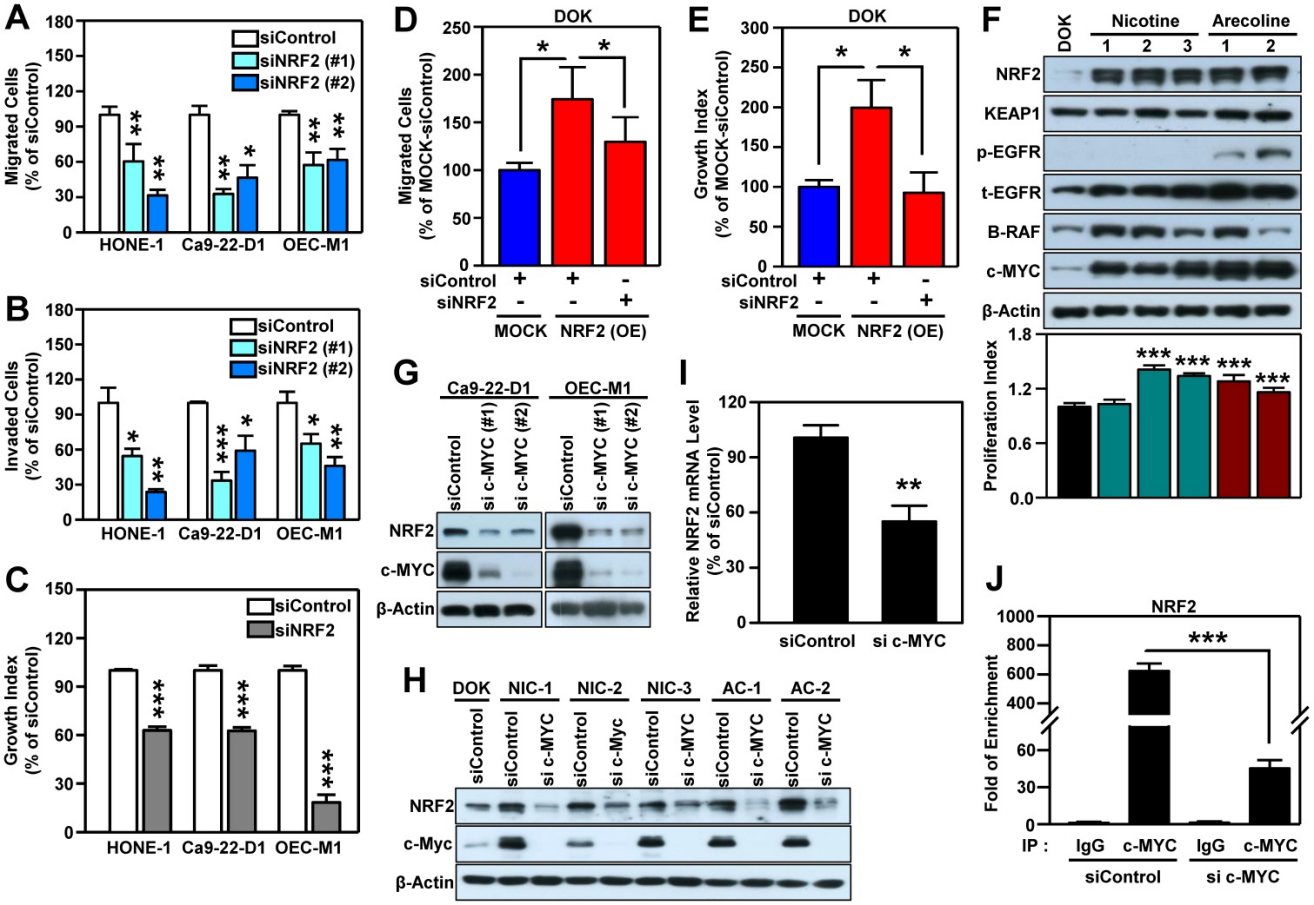
** NRF2 promotes malignant features of HNSCC *in vitro*.** Three human HNSCC cell lines, HONE-1, Ca9-22-D1 and OECM-1, were transiently transfected with NRF2 siRNA (siNRF2 #1 and siNRF2 #2) or scramble control (siControl). The motility and invasiveness of the transfected cells were evaluated by trans-well migration (A) and invasion (B) assays. The cells that had migrated or invaded were quantified and expressed as a percentage relative to siControl group. (C) The effects of NRF2-knockdown on cell growth of HNSCC cells. Cell number was determined using the methylene blue assay. (D) Migration ability was determined in DOK cells stably overexpressing NRF2 that were treated with NRF2 siRNA or a non-targeted control. To present relative cell migration, the value from the vector-only control DOK cells (blue bar) was set at 100%. (E) The growth rates of DOK cells stably overexpressing NRF2 and treated with NRF2 siRNA or a non-targeted control under low serum conditions were measured. The data were expressed as the percentage relative to the vector-only control DOK cell (MOCK) which was treated with negative control siRNA (blue bar). (F) Alteration of cellular properties with long-term exposure to nicotine or arecoline in dysplastic DOK cells. The nicotine- and arecoline-transformed DOK cells were established from DOK cells treated with non-toxic concentrations of carcinogens (nicotine: 500 µM; arecoline: 50 µM) for 1 year. The levels of NRF2, KEAP1, *phospho*-EGFR (Tyr1068), EGFR, B-RAF, and c-MYC in total lysates were assessed using Western blot analysis. The cell proliferation rates were measured by methylene blue assay after 72 h incubation and calculated as the fold increase compared to proliferation of the parental DOK cells (black bar). *Top*: representative Western blots. *Bottom*: the cell proliferation index. The effects of knockdown of *MYC* on NRF2 protein level in HNSCC cells (G) and carcinogen-transformed DOK cells (H). (I) RT-qPCR analysis of NRF2 mRNA levels in MYC-knockdown OEC-M1 cells. (J) ChIP-qPCR analysis of c-MYC binding at NRF2 promoter in MYC-knockdown OEC-M1 cells. All data are expressed as the mean ± S.D. from three individual experiments. * *p* < 0.05; ** *p* < 0.01; *** *p* < 0.001. vs. control.

To confirm that NRF2 drives the malignant progression of HNSCC, we knocked down the *NRF2* gene using RNA interference in three HNSCC cell lines (HONE-1, Ca9-22-D1, and OEC-M1; **Figure S4**) and found that both transient (**Figure 2A**) and stable (**Figure S5A-B**) knockdown of NRF2 markedly reduced cell migratory, invasive (**Figure 2B**), and growth (**Figure 2C**, **Figure S5C**) capacity compared to control. In contrast, stably overexpressed NRF2 facilitates cellular motility and growth; however, when NRF2 is re-suppressed, the migration and growth abilities were decreased in NRF2 overexpressing DOK cells (**Figure 2D-E**). Furthermore, by performing the soft agar colony formation assay, we found that knockdown of NRF2 significantly suppressed the ability of anchorage-independent growth in HNSCC cells (**Figure S5D**) and *vice versa* in NRF2 overexpressing cells (**Figure S5E**). In addition, the commercially available NRF2 inhibitor ML-385 produced results similar to genetic manipulation (**Figure S6**). These* in vitro* findings suggest that NRF2 is important for maintaining the malignant characteristics of HNSCC.

Next, we questioned whether carcinogenic substances modulate NRF2 during head and neck carcinogenesis. Cigarette smoking, alcohol consumption, and betel quid chewing are the leading risk factors for HNSCC 42-44. IHC staining showed that HNSCC patients who smoke, drink, and chew betel nut have higher nuclear NRF2 levels than those who do not have those habits (**Table S6**). Therefore, we used nicotine and arecoline to further investigate the role of NRF2 in HNSCC, as long-term nicotine (e.g., cigarette smoking) and arecoline (e.g., betel nut chewing) exposure have been reported to promote head and neck carcinogenesis and progression 28, 45, 46. We found that long-term exposure to nicotine or arecoline increased NRF2 expression, which was accompanied by an increased proliferation index in carcinogen-transformed DOK cells (**Figure 2F**). We previously established a 4-NQO (simulation of smoking) plus arecoline (simulation of betel nut chewing) induced mouse model of oral carcinogenesis 47, which mimics the pathogenesis of squamous cell carcinoma (SCC) in patients from Southeast Asia and Taiwan 48-50. For *in vivo* validation, we analyzed normal and SCC samples from tongue tissue from the 4NQO/arecoline co-induction oral SCC mouse model. The results demonstrated that expression and nuclear localization of NRF2 protein was increased in SCC compared to normal tongue tissue (**Figure S7**).

It is known that NRF2 is modulated by several oncogenes 20. By performing micro-western arrays, which enable quantitative, sensitive, and high-throughput assessment of protein abundance and modifications, we identified that BRAF, phospho-EGFR and c-MYC are significantly increased in both nicotine (NIC)- and arecoline (AC)-transformed DOK cells (**Figure S8**). Further validation revealed that increased expression levels of BRAF and c-MYC were observed in most carcinogen-transformed DOK cells (**Figure 2F**). Thus, we investigated whether BRAF or c-MYC were upstream modulators to upregulate NRF2 in HNSCC cells. As shown in **Figure S9**, we found silencing BRAF did not suppress NRF2 level in HNSCC cells. On the other hand, knockdown of *MYC* gene significantly inhibits NRF2 expression (**Figure 2G**). Similar results were observed in both nicotine (NIC)- and arecoline (AC)-transformed DOK cells (**Figure 2H**).

It is noteworthy that increased and differential *MYC* expression was observed in tumors of relevant clinical cancer datasets (**Table S5**). Therefore, we further explored the relationship between NRF2 and c-MYC. In addition to DOK cells, we confirmed the positive correlation between the expression of NFE2L2 (NRF2) and MYC in long-term arecoline-transformed normal human oral keratinocytes (NHOK) (**Figure S10**). Next, we determined whether NRF2 could regulate the expression of c-MYC and found that knockdown of NRF2 does not affect the expression of c-MYC protein in HNSCC cells (**Figure S11**). To investigate how c-MYC regulates NRF2 expression, we performed RT-qPCR analysis, and found that knockdown of c-MYC significantly decreased NRF2 mRNA levels in HNSCC cells (**Figure 2I**). Furthermore, we confirmed that c-MYC binds to the NRF2 promoter, and knockdown of c-MYC significantly reduced the binding strength of c-MYC at the NRF2-promoter in HNSCC cells (**Figure 2J**). These results indicate that c-MYC is involved in directing NRF2 expression in head and neck cancer.

### NRF2 drives tumor progression of HNSCC *in vivo*

**Figure 3:**
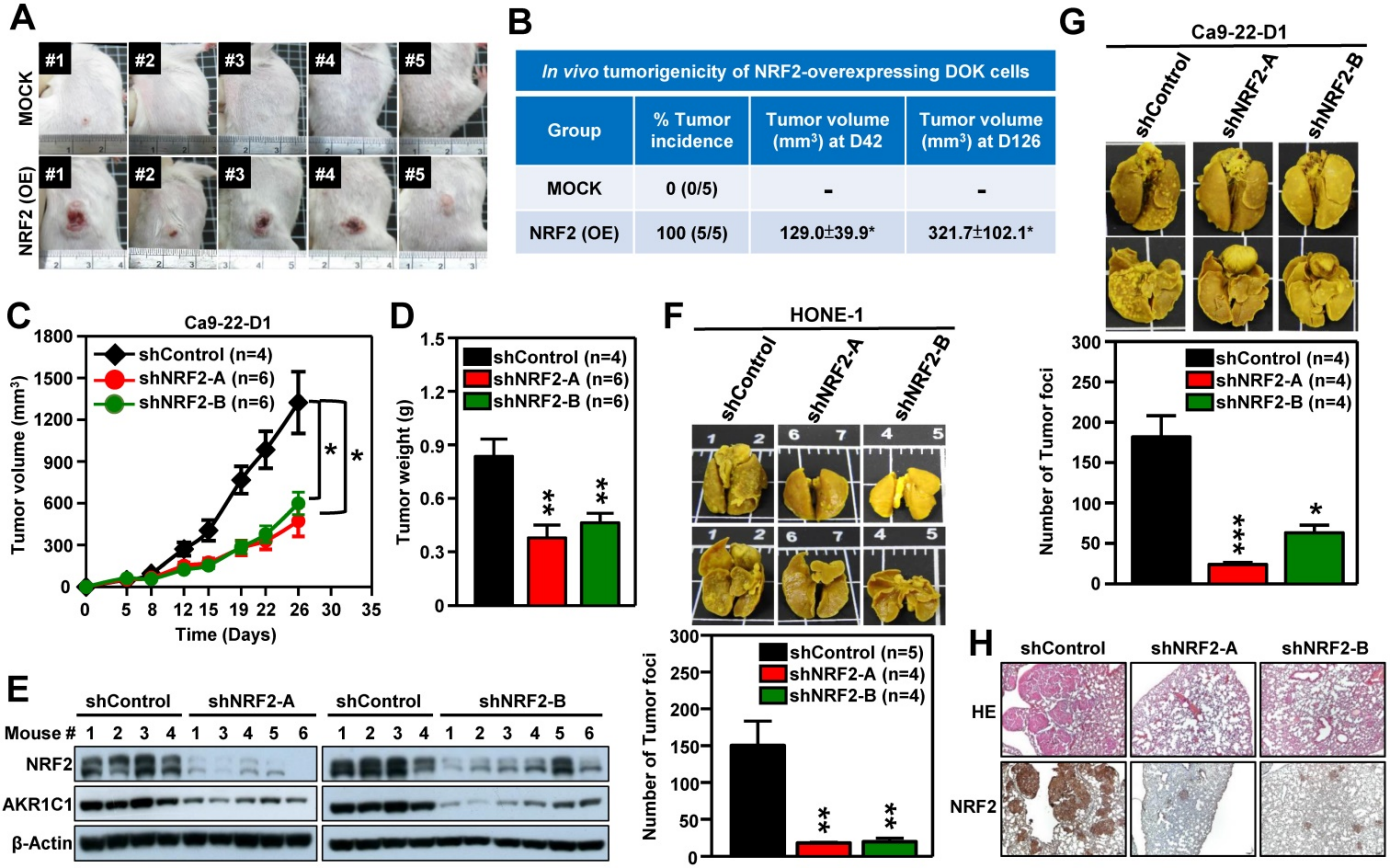
** NRF2 promotes tumorigenesis and malignant progression of HNSCC *in vivo*.** (A, B)* In vivo* tumorigenicity of NRF2 overexpressing dysplastic oral keratinocytes. DOK cells with stably overexpressed NRF2 (NRF2-OE) or vector-only control (MOCK) were subcutaneously injected into NOD/SCID mice (5×10^6^ cells per mouse), and tumor volume and body weight were measured weekly. (A) Representative photographs of mice with control (*upper*) and NRF2-overexpressing (*lower*) xenograft tumors. (B) The tumor incidence was defined as the percentage of mice in each group with a tumor volume that exceeded 50 mm^3^ in each group. Mean tumor volumes (mm^3^) of mice at days 42 and 126 were represented as mean ± S.E. (C, D) *In vivo* tumor growth assay of stable NRF2-knockdown Ca9-22-D1 cells. Tumor growth rate (C) and tumor weight (D) were compared between shControl (black) and two different shNRF2 groups (red and green). Mice were injected subcutaneously with Ca9-22-D1 cells. The tumor growth curves represent tumor volume data measured twice per week throughout the experiment. Mice were sacrificed and tumors were isolated and weighed at Day 26 after cell injection. (E) Expression of NRF2 and AKR1C1 was measured by Western blot assays of excised tumors from Ca9-22-D1 xenograft tumor-bearing mice. (F, G) *In vivo* experimental pulmonary metastasis assay of stable NRF2-knockdown HNSCC cells. HONE-1 (F) or Ca9-22-D1 (G) cells stably expressing NRF2-shRNA or control vector were delivered via tail vein injection into NOD/SCID mice. After 50 (HONE-1) or 25 (Ca9-22-D1) days, lungs were removed from all mice and fixed in Bouin's solution. The lung nodules were counted, and lung sections were prepared. *Upper panel:* Representative images of pulmonary metastatic foci produced after intravenous injection. *Lower panel:* Numbers of detectable tumor foci on the surface of whole lungs were quantified and indicated as mean ± S. E. * *p* < 0.05; ** *p* < 0.01; and *** *p* < 0.001 compared with the control group (shControl). (H) Representative histological photographs showing H&E staining and IHC analyses of lung sections taken under bright-field at 50× magnification using an upright microscope.

We next performed *in vivo* experiments to substantiate whether aberrant activation of NRF2 enhanced malignant progression of HNSCC. Firstly, we generated DOK cells with stable overexpression of NRF2 to examine the role of NRF2 in promoting malignant transformation *in vivo*. The pre-malignant epithelial cell line DOK is derived from human dorsal tongue tissue and is non-tumorigenic in mice 40. There was no tumor formation after implantation of mock control DOK cells *in vivo* (**Figure 3A**, *upper panel*). In contrast, implantation of DOK cells with stable overexpression of NRF2 resulted in tumor development *in vivo* (**Figure 3A**, *lower panel*). The tumor incidences of the NRF2-overexpressing and mock control groups were 100% and 0%, respectively (**Figure 3B**). In our next experiment, we investigated how NRF2 expression could affect tumor growth using the Ca9-22-D1 (human origin) xenograft tumor model. As shown in **Figure 3C-D**, tumor growth was inhibited in mice bearing stable NRF2-knockdown Ca9-22-D1 xenograft tumors. In addition, we found that the expression levels of NRF2 and AKR1C1 (the classical NRF2 downstream target) proteins were significantly reduced in NRF2-knockdown tumors compared to controls (**Figure 3E**). To evaluate whether the depletion of NRF2 suppresses metastasis of HNSCC *in vivo*, we used an experimental metastasis model by injecting mice with HONE-1 or Ca9-22-D1 cells, either with stably expressing NRF2-shRNA or empty expression vector. Compared with the mock control group, a decrease in lung-specific metastatic foci formation was observed in NRF2-knockdown groups (**Figure 3F-G**), suggesting that genetic ablation of NRF2 remarkably hindered pulmonary metastasis. The hematoxylin and eosin (H&E) and IHC staining of lung tissue sections demonstrated that mice bearing NRF2-knockdown Ca9-22-D1 cells significantly inhibited lung-specific metastatic tumor foci formation compared with mice bearing mock control cells, and NRF2 expression level is positively correlated with the number of tumor foci (**Figure 3H**). These results suggest that augmentation of NRF2 expression drastically enhances the potential for malignant transformation, tumor growth, and metastasis of HNSCC *in vivo*.

### NRF2-mediated antioxidant capacity is not the primary cause of malignant progression of HNSCC

**Figure 4:**
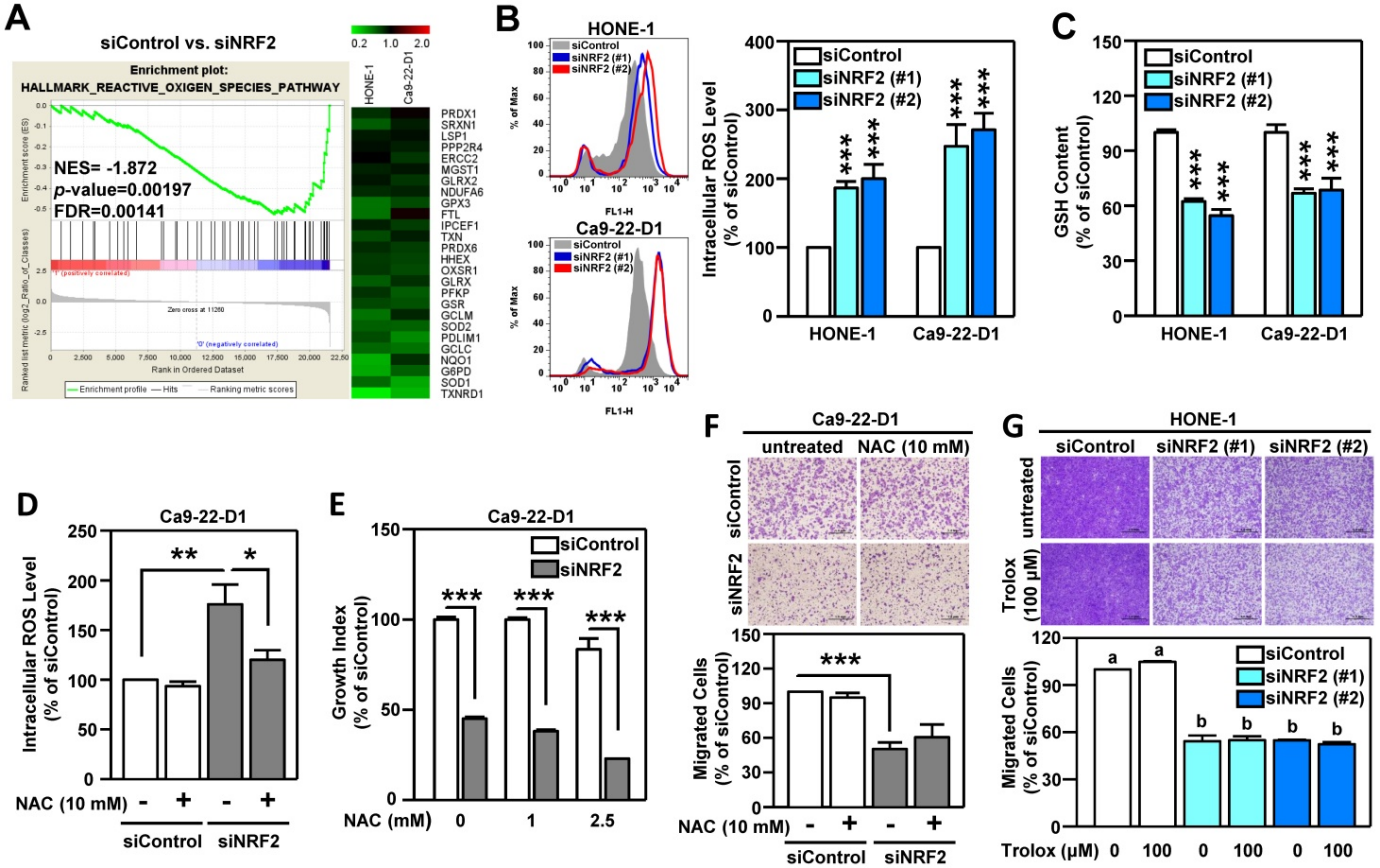
** NRF2-mediated antioxidant defense is not a primary cause of malignant progression of HNSCC cells.** (A) GSEA enrichment plots of the HALLMARK REACTIVE OXYGEN SPECIES PATHWAY and the corresponding heat map for siControl versus siNRF2 groups in HONE-1 and Ca9-22-D1 cells. (B, C) NRF2 modulates ROS and glutathione. The intracellular ROS level (B) and reduced form of glutathione (GSH; C) levels were measured in NRF2 knockdown HNSCC cell lines by fluorescent staining of 2', 7'-dichlorodihydrofluorescein (DCF) and monochlorobimane (MBC)-GSH adducts, respectively. (D) NRF2-knockdown Ca9-22-D1 cells were assayed for intracellular ROS levels in the absence and presence of 10 mM N-acetylcysteine (NAC). The relative intracellular ROS levels were normalized to the siControl group without NAC treatment. The effects of elimination of ROS by NAC on cell survival (E) and migration (F) in NRF2-knockdown Ca9-22-D1 cells. (G) The effects of elimination of ROS by Trolox (100 μM) on cell migration in NRF2-knockdown HONE-1 cells. All data are expressed as the mean ± S.D. from three individual experiments. Groups with *different letters* are significant different (*p* < 0.05) from each other. * *p* < 0.05; ** *p* < 0.01; *** *p* < 0.001.

Oxidative stress is known to play a pivotal role in the development of HNSCC 51. The primary function of NRF2 is to maintain cellular redox homeostasis through enhancing antioxidant gene expression. Therefore, we investigated whether the change in redox balance is the primary cause of NRF2-mediated malignant progression in HNSCC. Gene Set Enrichment Analysis (GSEA) revealed that the HALLMARK REACTIVE OXYGEN SPECIES pathway genes were highly enriched in NRF2-silenced HNSCC cells and that there was a concomitant decrease in the expression of a panel of NRF2-mediated antioxidant genes (**Figure 4A**). Profoundly increased intracellular ROS levels were also found in both Ca9-22-D1 and HONE-1 cells (**Figure 4B**). In addition, DOK cells with stable overexpression of NRF2 exhibited lower ROS levels than mock controls (**Figure S12A**). When cells were challenged with oxidative stress, the reduced form of glutathione (GSH) was oxidized to form glutathione-disulfide (GSSG), which brought on a decrease in intracellular GSH levels. Knockdown of NRF2 significantly reduced intracellular GSH levels in HNSCC cells (**Figure 4C**). Although treatment of cells with N-acetyl cysteine (NAC), a widely used pharmacological antioxidant, effectively reduced ROS production (**Figure 4D**, **Figure S12B**), addition of NAC did not restore cell growth (**Figure 4E**, **Figure S12C**) and migration ability (**Figure 4F**, **Figure S12D**) in NRF2-knockdown HNSCC cells. Similarly, treatment with another antioxidant, Trolox, had no effect in the rescue of cell motility in NRF2-knockdown HNSCC cells (**Figure 4G**). These results suggested that NRF2-mediated antioxidant capacity was not a primary cause of malignant progression of HNSCC.

### Pentose phosphate pathway (PPP) is the most highly enriched NRF2-mediated metabolic pathway in HNSCC cells

**Figure 5:**
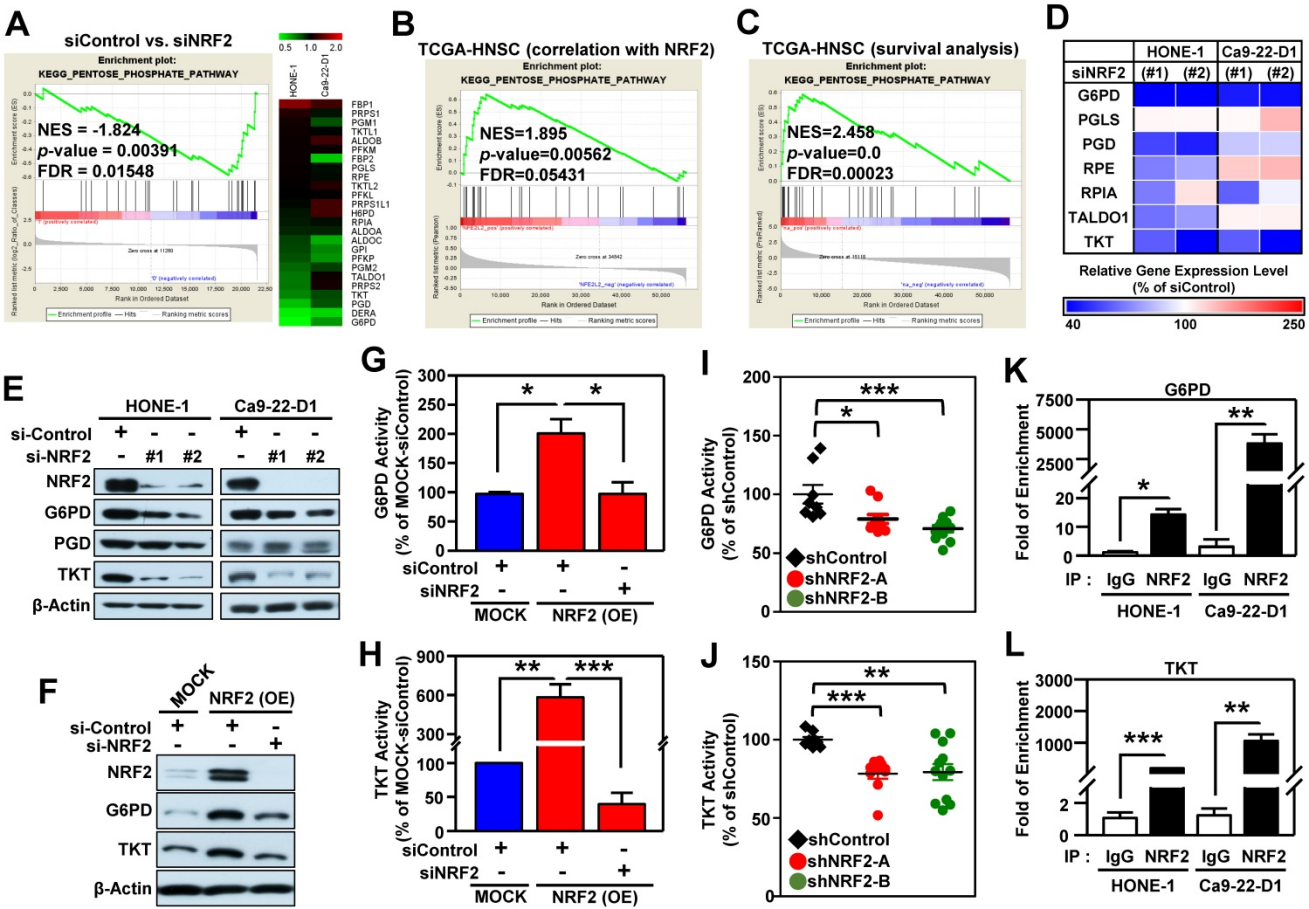
** The pentose phosphate pathway is altered in NRF2-knockdown HNSCC cells.** (A) GSEA enrichment plots and heat maps of differentially expressed genes belonging to the KEGG PENTOSE PHOSPHATE PATHWAY (PPP) and associated with knockdown of NRF2 in HNSCC cells. (B) GSEA of the TCGA-HNSC dataset showed that NRF2 level was positively correlated with the PPP pathway. (C) GSEA demonstrated that upregulation of the PPP was associated with poor survival of HNSCC according to gene expression and overall survival data in the TCGA-HNSC dataset. (D) Heat-map of relative gene expression, assessed via real-time PCR, in NRF2-knockdown cells compared to the non-targeted negative control. Gene expression in the NRF2-knockdown cells was significantly different (*p* < 0.05) compared to scramble control and is represented as the average value of three individual experiments per heat map square (siControl = 100%). (E) Total G6PD and TKT protein levels were assessed in NRF2-knockdown HONE-1 and Ca9-22-D1 cells by Western blot. β-Actin was detected as a loading control. (F) G6PD and TKT protein levels were assessed in NRF2-overexpressing DOK cells treated with NRF2 siRNA or non-targeted control. Examination of the changes of G6PD (G) and TKT (H) enzyme activities in NRF2-overexpressing DOK cells treated with NRF2 siRNA or non-targeted control. G6PD (I) and TKT (J) enzyme activity assays were performed on the excised tumors from Ca9-22-D1 xenograft tumor-bearing mice. ChIP-*q*PCR analysis of G6PD (K) and TKT (L) were performed on HONE-1 and Ca9-22-D1 cells by using normal rabbit IgG or an anti-NRF2 antibody, and the result was normalized to input control values and represented as the fold enrichment relative to the anti-normal rabbit IgG control. All data are expressed as the mean ± S.D. from three individual experiments. * *p* < 0.05; ** *p* < 0.01; *** *p* < 0.001.

After a close examination of GSEA data, we noticed that 33% (10/30) of the top 30 most highly enriched down-regulated gene sets in NRF2-knockdown cells were related to cell metabolism (**Figure S13A**). Among them, the highest-ranking metabolic pathway is the KEGG PENTOSE PHOSPHATE PATHWAY (PPP; **Figure S13B**). The enrichment plot and heat map of differentially expressed genes displayed obvious suppression of PPP in NRF2-knockdown HONE-1 and Ca9-22-D1 cells (**Figure 5A**). Since the PPP is a major source of reduced nicotinamide adenine dinucleotide phosphate (NADPH), we checked and observed that NADPH was also significantly suppressed upon NRF2 inhibition (**Figure S14**).

To explore which biological processes or molecular pathways could be regulated by NRF2 signaling in the milieu of tumors, we again performed GSEA analysis on head and neck tumor samples annotated in The Cancer Genome Atlas Head-Neck Squamous Cell Carcinoma (TCGA-HNSC) dataset. We queried significant enrichment of gene sets that were co-expressed with* NRF2*. At nominal (NOM) *p-*value < 0.05 and FDR < 0.25, we found 511 gene sets were highly co-expressed with *NRF2*. As expected, PPP pathway also showed significant positive enrichment (NES = 1.895) among these gene sets, indicating a positive correlation between PPP genes and NRF2 level (**Figure 5B**). We conducted another independent GSEA assay based on Cox regression model to query whether any enrichment of genes was associated with clinical outcomes of HNSCC. Notably, the PPP pathway was found to be significantly associated with patient survival (NES = 2.458; **Figure 5C**). These results indicate that increased PPP activity may predict poor overall survival of the patients with HNSCC.

We next identified the specific PPP genes enriched in HNSCC by performing *q*PCR analysis, and the results demonstrated that G6PD, 6-phosphogluconate dehydrogenase (PGD), and TKT were significantly suppressed in NRF2-knockdown HONE-1 and Ca9-22-D1 cells (**Figure 5D**). Remarkably, knockdown of NRF2 drastically suppressed the protein levels and enzymatic activity of G6PD and TKT in HNSCC cells (**Figure 5E**, **Figure S15A-B**). Consistently, the protein levels or enzyme activity of G6PD and TKT were increased in stable NRF2-overexpressing cells (**Figure 5F-H**,**Figure S15C**) and could be re-suppressed by NRF2-targeted siRNA treatment (**Figure 5F-H**). Furthermore, the *in vivo* study revealed that knockdown of NRF2 markedly decreased the protein levels (**Figure S15D**) and enzymatic activities (**Figure 5I-J**) of G6PD and TKT in the excised tumors from Ca9-22-D1 xenograft tumor-bearing mice.

We further dissected the mechanisms by which NRF2 regulates G6PD and TKT in HNSCC cells. ChIP-*q*PCR assays demonstrated direct binding of NRF2 to the ARE consensus sequence in the promoter region of G6PD and TKT in both HONE-1 and Ca9-22-D1 cells (**Figure 5K-L**). Analysis of data from the TCGA-HNSC cohort demonstrated that the level of G6PD or TKT gene expression was positively correlated with NRF2 (G6PD vs. NRF2: Pearson correlation = 0.49, *p* < 4.7e-33; TKT vs. NRF2: Pearson correlation = 0.36, *p* < 2.7e-17; **Figure S16**). These results indicated that NRF2 repurposes PPP metabolism by direct modulation of G6PD and TKT in head and neck cancer.

### G6PD and TKT are key NRF2-downstream effectors to drive malignant progression of HNSCC

**Figure 6:**
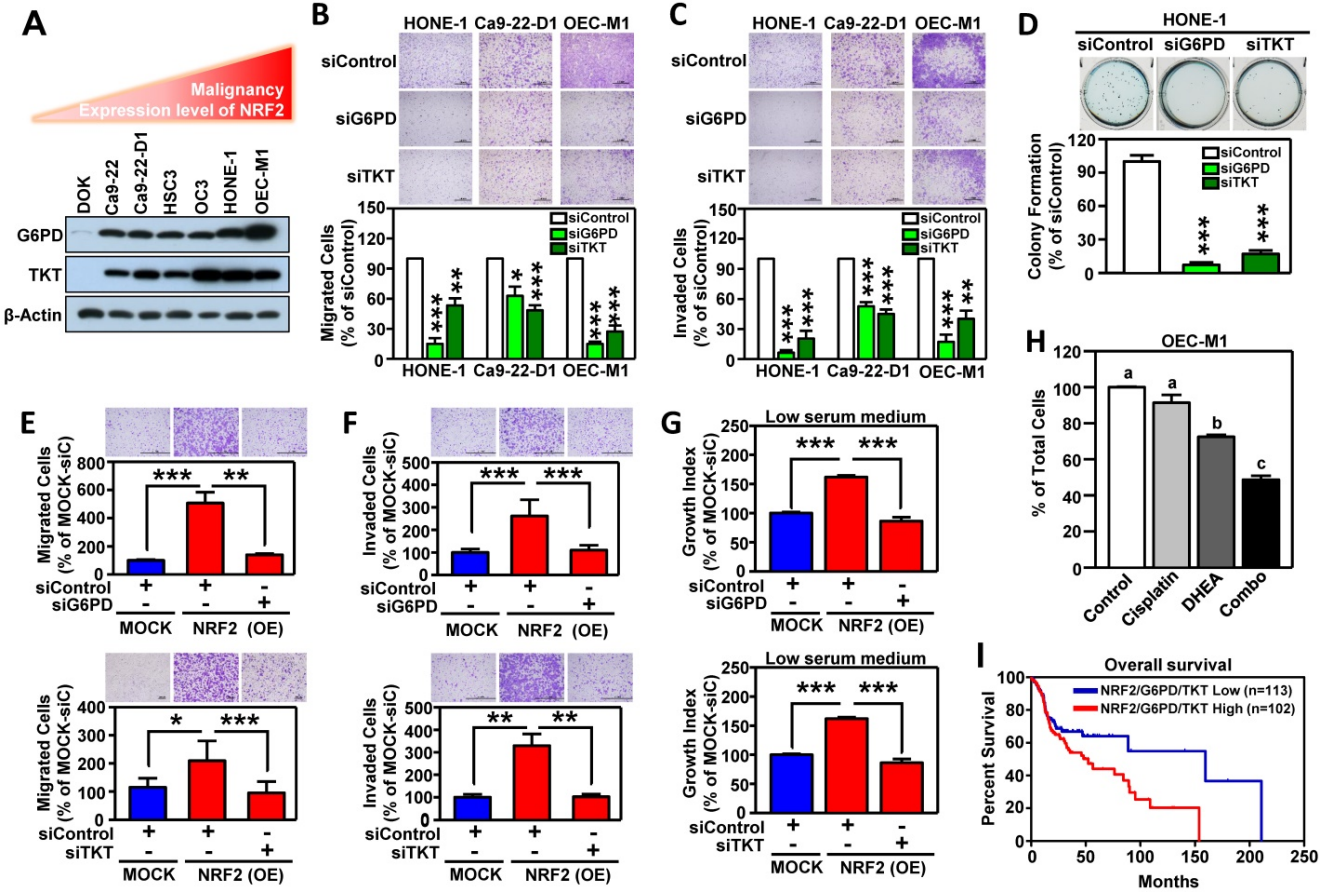
** G6PD and TKT are downstream effectors of NRF2-mediated malignant progression of HNSCC.** (A) Expression patterns of G6PD and TKT were positively related to malignancy and NRF2 protein level in HNSCC cells. (B) Cell motility and (C) invasiveness were inhibited in G6PD- and TKT-knockdown HNSCC cells. Three different HNSCC cell lines, HONE1, Ca9-22-D1, and OEC-M1, were transiently transfected with siRNA against G6PD (siG6PD), TKT (siTKT), or with a non-targeted control siRNA (siControl) for 48 h, resulting in down-regulation of G6PD and TKT expression. The motility (B) and invasiveness (C) of G6PD- or TKT-knockdown HNSCC cells were evaluated by trans-well migration or invasion assays. (D) Anchorage-independent growth of G6PD- and TKT-knockdown HONE-1 cells was evaluated by soft agar colony formation. Silencing of the *G6PD* or *TKT* genes by siRNA diminished cell migration (E), invasion (F), and proliferation (G) of NRF2-overexpressing DOK cells. (H) Inhibition of G6PD induced synergistic cytotoxicity with cisplatin in HNSCC cells. Ca9-22-D1 cells were treated with the G6PD inhibitor DHEA, cisplatin, or a combination of the two agents for 72h. Cell viability was evaluated via the methylene blue assay and presented as the percentage of viable cells compared to the control group (white bar). Groups with *different letters* had significantly different percent viability (*p* < 0.05). (I) Kaplan-Meier curves demonstrated correlation between high NRF2/G6PD/TKT expression and poor overall survival of HNSCC patients via analysis of the TCGA dataset. The data are expressed as the mean ± S.D. from three individual experiments. * *p* < 0.05; ** *p* < 0.01; *** *p* < 0.001.

We next investigated whether G6PD and TKT were critical downstream effectors in driving NRF2-mediated malignant progression of HNSCC. First, we found that the levels of G6PD and TKT expression (**Figure 6A**) were positively correlated with hallmarks of cell malignancy (i.e., motility as shown in **Figure 1F**). As expected, knockdown of G6PD or TKT lead to suppression of cell migration, invasion, and anchorage-independent growth of HNSCC cells (**Figure 6B-D**). We also observed that increase of NRF2 drives cell motility, invasiveness, and proliferation in stably NRF2-overexpressing DOK cells, and these effects could be re-suppressed after blocking the function of G6PD and TKT (**Figure 6E-G**). These results indicated that G6PD and TKT are key NRF2-downstream effectors to drive malignant features of HNSCC.

The combination of pathway targeted therapies with standard-of-care (S.O.C.) treatment is a promising therapeutic strategy that may generate synthetic lethality 52. Cisplatin concurrent chemoradiotherapy (CCRT) is a S.O.C. therapy for treatment of HNSCC, therefore, we questioned whether targeted G6PD or TKT could elicit a synergistic interaction with cisplatin. We treated HNSCC cells with either low dose cisplatin alone, or in combination with G6PD or TKT inhibitors. Strikingly, three different G6PD inhibitors, including dehydroepiandrosterone (DHEA) 53, *trans*-polydatin 54, and 6-aminonicotinamide (6-AN) 55 were found to cause synthetic lethality in HNSCC cells when combined with cisplatin (**Figure 6H**, **Figure S17**), while co-treatment with the TKT inhibitor (oxythiamine chloride hydrochloride) and cisplatin showed no synergistic cytotoxicity (data not shown). These results indicate that combined treatment with cisplatin plus a G6PD inhibitor may be particularly effective as a synthetic lethal therapeutic regimen for HNSCC, and clinical application of this strategy merits further investigation. Although the combination of a TKT inhibitor and cisplatin did not show synergy in HNSCC cells, targeting TKT may have a synergistic interaction with other approved drugs; further research is needed to explore additional combinations.

To expand the level of evidence, the clinical outcome of NRF2/G6PD/TKT-gene signature in HNSCC patients was analyzed in TCGA-HNSC cohort. A total of 215 patient samples were divided into the NRF2/G6PD/TKT-high group (red; n = 102) and the NRF2/G6PD/TKT-low group (blue; n = 113) based on gene expression patterns. The survival probability estimates for the two groups were visualized as Kaplan-Meier plots. Notably, overall survival analysis revealed that the patients in the NRF2/G6PD/TKT-high (red) group had worse survival outcomes than the NRF2/G6PD/TKT-low (blue) group (*p* = 0.05; **Figure 6I**). These results suggested that the NRF2/G6PD/TKT gene set could be a potential prognostic biomarker to predict the clinical outcome of HNSCC.

### Ribose-5-phosphate generation is more important than NADPH production in determining malignant features of head and neck cancer

**Figure 7:**
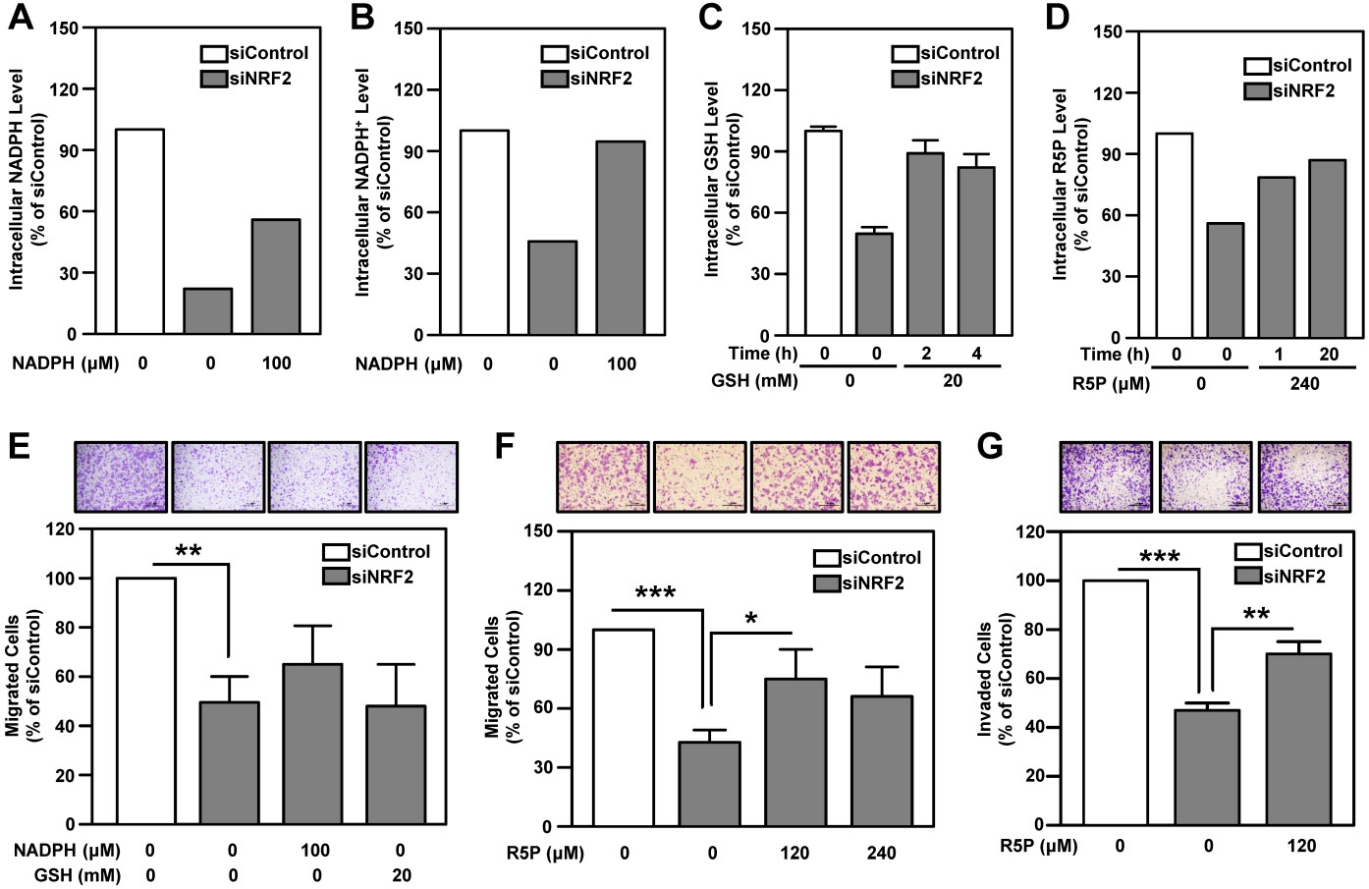
** The role of PPP-related metabolites in rescuing cell motility and invasiveness of NRF2-knockdown HNSCC cells.** Effects of NADPH supply on intracellular NADPH (A) and NADP^+^ (B) levels in NRF2-knockdown Ca9-22-D1 cells. Intracellular NADPH and NADP^+^ levels were measured after incubation with or without 100 μM NADPH for 4 h. (C) Effects of GSH supply on intracellular GSH levels in NRF2-knockdown Ca9-22-D1 cells. Intracellular GSH was measured after incubation with or without 20 mM GSH for 2 h or 4 h. (D) Effects of R5P supply on intracellular R5P in NRF2-knockdown Ca9-22-D1 cells. Intracellular R5P was measured after incubation with or without 240 μM R5P for 1 h or 20 h. Intracellular metabolite levels were normalized to those of the respective siControl without treatment. (E) The effects of NADPH and GSH on cell migration ability in NRF2-knockdown Ca9-22-D1 cells. Cell motility (F) and invasiveness (G) were determined by trans-well migration or invasion assay in NRF2-knockdown Ca9-22-D1 cells in the presence or absence of R5P. The data are expressed as the mean ± S.D. from three individual experiments. * *p* < 0.05; ** *p* < 0.01; *** p < 0.001.

NADPH and ribose 5-phosphate (R5P) are the main products or intermediates of G6PD and TKT. NADPH in cells maintains glutathione in the reduced state (GSH) and is important for reductive biosynthesis (e.g., fatty acid synthesis). R5P is required for de novo nucleotide biosynthesis 56. The metabolite analysis showed that intracellular GSH, NADPH, NADP^+^ and nucleotide metabolism-related metabolites, especially R5P, were down-regulated in NRF2-knockdown HNSCC cells (**Table S7**). We found that replenishment of metabolites can effectively rescue the levels of NADPH, NADP^+^, GSH and R5P in NRF2-knockdown cells (**Figure 7A-D**). However, addition of NADPH and GSH does not significantly restore cell motility in NRF2-knockdown HNSCC cells (**Figure 7E**). Notably, unlike NADPH and GSH, R5P significantly restored cell motility and invasiveness in NRF2-knockdown HNSCC cells (**Figure 7F-G**;**Figure S18**).

Nucleotide biosynthesis begins with the conversion of R5P to 5-phosphoribosyl-1-pyrophosphate (PRPP), which is then catalyzed to become phosphoribosylamine, a nucleotide precursor. Therefore, we performed a rescue experiment by adding PRPP to the growth media of NRF2-knockdown HNSCC cells. The result demonstrated that, similar to R5P, PRPP could rescue the cell migration ability under NRF2 knockdown condition (**Figure S18**). These results suggested that the regulatory function of G6PD/TKT on nucleotide biosynthesis appears to be more important than redox regulation for malignant progression of head and neck cancer.

## Discussion

In HNSCC, activation of NRF2 occurs through gain-of-function mutations in *NFE2L2* (gene encoding NRF2) 57-59, loss-of-function mutations in the* KEAP1* gene 57, disruption of the KEAP1/CUL3/RBX1 E3-ubiquitin ligase complex 31, modulation by IL-6 18 or GRP78/PERK signaling 19, and increased protein stability of NRF2 via *O*-GlcNAcylation 23. In this study, we are the first to provide evidence that aberrant activation of NRF2 is modulated by c-MYC in chemical-induced head and neck tumorigenesis. Major chemical-associated risk factors for development of HNSCC, including alcohol consumption, betel nut chewing, and cigarette smoking, all generate oxidative stress 60. NRF2 is a key determinant for cells coping with oxidative stress and a tobacco exposure-related signature in HNSCC 57, 61. Long-term exposure of dysplastic DOK cells to nicotine (simulating smoking) or arecoline (simulating betel nut chewing) was used to mimic the malignant transformation *in vitro*, and the result demonstrated that increased cell proliferation was accompanied by increased NRF2, B-RAF, and c-MYC expression in carcinogen-transformed cells (**Figure 2F**, **Figure S8**). Through additional experiments we excluded B-RAF and confirmed that c-MYC positively modulates NRF2 through binding to the NRF2 promoter region in HNSCC cells (**Figure 2G-2J**). ChIP-seq data from the ENCODE consortium demonstrated that c-MYC binds directly to the* NFE2L2* locus and increases *NRF2* transcription 20. The *MYC* gene has been shown to be amplified in HNSCC, particularly in HPV(-) HNSCC 57. Our study demonstrated that long-term exposure of DOK and NHOK cells with carcinogens induces c-MYC expression (**Figure 2F**, **Figure S8** and **S10**), suggesting that chemical factors may be more important to initiate dysregulation of c-MYC oncogene than viral factors during head and neck carcinogenesis.

Through systematic investigation by combining RNAi, microarrays, and GSEA approach, we noted that 33% (10/30) of the 30 most highly enriched down-regulated gene sets in NRF2-knockdown HNSCC cells were related to cellular metabolism (**Figure S13**). Metabolic reprogramming is firmly established as a hallmark of cancer 62. During malignant development, cancer cells are obliged to rewire their metabolism to support aberrant cell growth, enhance their metastatic capacity, and adapt to the stress of survival in the austere tumor microenvironment 63. A characteristic feature of head and neck cancer is enhanced glycolysis, providing the basis for clinical use of FDG-PET as a diagnostic imaging agent 64. In addition, recent studies on NRF2-mediated cellular metabolism in HNSCC are focused on glycolysis (the Warburg effect). Chang *et al.* demonstrated that the Warburg effect and the stemness of cancer-initiating cells of the head and neck were mediated by GRP78/p-PERK/NRF2 signaling 19. Fu *et al.* found that hyperactive NRF2 causes metabolic reprogramming and up-regulation of glycolysis genes, such as pyruvate kinase M2 (PKM2), in the mouse esophagus 26. In this study, we observed that KEGG GLYCOLYSIS GLUCONEOGENESIS pathway is the fourth most highly enriched down-regulated gene set in NRF2-knockdown HNSCC cells (**Figure S13**), which mirrored previous reports 19, 26.

In addition to highlighting glycolysis, we have now identified the PPP pathway as the most highly enriched down-regulated metabolism gene set in NRF2-knockdown HNSCC cells (**Figure S13**). More importantly, as supported by clinical evidence, the expression of PPP genes is positively correlated with NRF2 (**Figure 5B**), and increased PPP predicted poor overall survival of patients with HNSCC (**Figure 5C**). PPP branches off from glycolysis and comprises irreversible oxidative and reversible non-oxidative phases. The enzymes involved in the oxidative phase are G6PD, 6-phosphogluconolactonase (PGLS), and PGD. Ribose-5-phosphate isomerase (RPI), ribulose 5-phosphate 3-epimerase (RPE), transaldolase (TALDO1), and TKT function in the non-oxidative phase of the PPP. To date, very little is known regarding these PPP enzymes in head and neck cancer. NRF2-regulated PPP genes, including G6PD, PGD, TKT, and TALDO1 were first identified in lung adenocarcinoma A549 cells 13. In this study, we confirmed that G6PD and TKT mRNA level, protein level, and enzyme activity were consistently regulated by NRF2 in HNSCC *in vitro* and *in vivo* (**Figure 5**, **Figure S15**).

G6PD is overexpressed in a number of cancer types 65, including HNSCC (**Table S5**). Several oncogenic pathways associated with PPP flux regulation are linked with G6PD 65. G6PD can be transcriptionally activated by TAp73 66, YY1 67, and NRF2 13. However, only one prior study revealed that G6PD was modulated by FOXM1 for participating radio-resistance in head and neck cancer 68. TKT had been reported to be involved in the malignant progression of breast cancer 69 and liver cancer 70, however, there is very limited information on TKT in HNSCC. Based on Oncomine database analysis, increased TKT expression was observed in tumors in several clinical HNSCC datasets (**Table S5**). It has been shown that transketolase like 1 (TKTL1), a TKT isoform, is activated by promoter hypomethylation and contributes to HNSCC carcinogenesis 71. TKT, TKTL1, and TKTL2 encoded enzymes all have transketolase activity. Our study showed that transketolase activity was significantly reduced in NRF2-knockdown HNSCC cells and xenograft tumors (**Figure 5**, **Figure S15**), and NRF2 did not affect the expression of *TKTL1* or *TKTL2* gene in HNSCC (data not shown). These results suggested that knockdown of NRF2-mediated TKT suppression did not cause compensatory effects by increasing TKTL1 and TKTL2. Knockdown of TKT has been reported to inhibit the proliferation of oral cancer cells and be accompanied by an increased uptake of glucose and glutamine, as well as increased production of lactate 72, which suggested that cancer cells may overcome single defective pathways through secondary metabolic network adaptations. Therefore, inhibition of multiple metabolic pathways or their common upstream regulator may be required to impede tumor progression. Accordingly, inhibition of NRF2 may be more advantageous compared to targeting a particular downstream metabolic pathway. Although there have yet to be any FDA-approved drugs targeting NRF2 activity in cancer, there have been increasing efforts toward the development of novel NRF2 inhibitors 73 and continued research in this area has great potential for clinical application.

NRF2 has been shown to activate PPP genes through direct binding to the ARE in the gene promoters 13, 21, producing effects such as attenuation of miR-1 and miR-206 12 or driving telomerase reverse transcriptase 74. We demonstrated that NRF2 bound directly to the ARE consensus sequence in the promoters of *G6PD* and *TKT* in HNSCC cells (**Figure 5K-5L**), and G6PD and TKT acted as the key NRF2-downstrem effectors of malignant progression of HNSCC (**Figure 6**). G6PD is the irreversible rate limiting enzyme in the oxidative phases of the PPP that ensures sufficient NADPH and R5P levels. The reversible enzyme TKT bridges the PPP with glycolysis, and is therefore a key enzyme controlling the direction of PPP and R5P levels 56. Although the requirement for NADPH is higher than that for R5P in cell physiological functions, the addition of R5P can restore cell motility in NRF2-knockdown HNSCC cells, whereas the addition of NADPH does not rescue motility (**Figure 7**). These results suggest that G6PD- and TKT-regulated nucleotide biosynthesis may be more important than redox regulation in driving malignant progression of head and neck cancer. NADPH could be generated by both PPP and the folate-mediated one-carbon metabolism and malic enzymes in cancer cells and other proliferating cells 75, 76, therefore, we speculate that inhibition of the PPP in HNSCC cells may reprogram other NADPH homeostasis systems. Additional research is needed to explore this possibility.

In fact, the mechanism by which NRF2 regulates nucleotide biosynthesis varies among cancer types. Saigusa *et al.* found that TALDO1 may involve in NRF2-mediated de novo purine nucleotide synthesis in non-small-cell lung carcinoma 77. However, we noted that in all test HNSCC cells, the expression level of the TALDO1 gene did not consistently change after genetic ablation of NRF2 (**Figure 5D**). In addition, Mitsuishi *et al.* has reported that MTHFD2, the metabolism enzyme for nucleotide biosynthesis, was decreased in lung cancer cell lines with NRF2 knockdown 13. DeNicola *et al.* demonstrated that NRF2 controls the expression of PHGDH, PSAT1, PSPH, and SHMT2 via ATF4 to support nucleotide production in non-small cell lung cancer 25. However, according to our real-time PCR validation, the expression levels of these genes (PHGDH, PSAT1, PSPH, SHMT1/2, MTHFD1/2) did not change upon NRF2 silencing in HNSCC cells (**Figure S19**), suggesting that NRF2-mediated regulatory mechanisms may vary substantially in head and neck versus lung cancer, further supporting the unique role of NRF2-mediated G6PD and TKT expression in nucleotide biosynthesis of head and neck cancer. More importantly, we have identified the NRF2/G6PD/TKT gene signature as a potential prognostic biomarker for prediction of HNSCC survival outcomes (**Figure 6I**). Even though it was known that NRF2 can regulate PPP 12, 13, 21, no study has demonstrated that NRF2 promotes cancer progression directly through PPP components, particularly in head and cancer. In this study, we provided the first evidence to confirm that G6PD and TKT are critical downstream effectors of NRF2-driven malignant progression in HNSCC. We believe this is an important breakthrough for clarifying the role of NRF2 in cancer development.

In addition to PPP and glycolysis, the ten most highly enriched down-regulated metabolism pathways in NRF2 knockdown HNSCC cells also included amino acid, nucleotide, xenobiotics, heme, and fatty acid metabolism as well as oxidative phosphorylation (**Figure S13B**), and many of them have been known to modulate head and neck oncogenesis 78. Interestingly, we noted that the KEGG VALINE, LEUCINE AND ISOLUCINE DEGRADATION pathway was the second most highly enriched downregulated gene set in response to NRF2 silencing in HNSCC cells. Valine, leucine, and isoleucine are branched-chain amino acids (BCAAs). BCAA metabolism can influence diverse cellular processes, ranging from protein synthesis to epigenetic regulation, and dysregulation of BCAA metabolism contributes to cancer progression by diverse mechanisms 79. To date, no literature has been published on the regulation between NRF2 and BCAA metabolism. In addition, the role of valine, leucine, and isoleucine degradation in the progression of head and neck cancer remains to be clarified. Further exploration of the link between BCAA metabolism and NRF2 in HNSCC is warranted.

This is the first study providing data from systematic analysis of the regulatory mechanisms and function of NRF2 in HNSCC. We discovered that aberrant activation of NRF2 occurred via c-MYC-mediated upregulation and is associated with chemical-induced carcinogenesis. NRF2 is involves in reprogramming the broad metabolic milieu in HNSCC, and PPP is the most highly enriched NRF2-mediated metabolic pathway. Notably, G6PD and TKT are critical NRF2-downstream effectors that drive malignant progression of HNSCC. Furthermore, G6PD- and TKT-regulated nucleotide biosynthesis is more important than redox regulation for maintaining the malignant features of head and neck cancer. Taken together, these data support the potential for use of the NRF2/G6PD/TKT gene signature as a prognostic biomarker and for the development of NRF2-targeted therapies that alter cellular metabolism as novel and promising treatment options for head and neck cancer.

## Abbreviations

AC: arecoline
ARE: antioxidant response element
BCAAs: branched-chain amino acids
CCRT: cisplatin concurrent chemoradiotherapy
DHEA: dehydroepiandrosterone
DOK: human dysplastic oral keratinocytes
F6P: fructose 6-phosphate
G3P: glyceraldehyde 3-phosphate
G6P: glucose 6-phosphate
G6PD: glucose-6-phosphate dehydrogenase
GSH: glutathione
GSSG: glutathione-disulfide
HNSCC: head and neck squamous cell carcinoma
KEAP1: Kelch‐like ECH associating protein 1
NAC: N-acetyl cysteine
NHOK: normal human oral keratinocytes
NIC: nicotine
NRF2: nuclear factor erythroid 2-related factor 2
OT: oxythiamine chloride hydrochloride
PGD: 6-phosphogluconate dehydrogenase
PGLS: 6-phosphogluconolactonase
PKM2: pyruvate kinase M2
PPP: pentose phosphate pathway
PRPP: 5-phosphoribosyl-1-pyrophosphate
R5P: ribose 5-phosphate
ROS: reactive oxygen species
RPE: ribulose 5-phosphate 3-epimerase
RPI: ribose-5-phosphate isomerase
S.O.C.: standard-of-care
TALDO1: transaldolase
TKT: transketolase
TKTL1: transketolase like 1
