# Supplementary material for: c-MYC-directed NRF2 drives malignant progression of head and neck cancer via glucose-6-phosphate dehydrogenase and transketolase activation
Source: Theranostics. 2021 Mar 11;11(11):5232–47. doi: 10.7150/thno.53417 (PMC8039948; doi:10.7150/thno.53417)
Supplement: Supplementary file 1 — Supplementary figures and tables. [file thnov11p5232s1.pdf]

c-MYC-directed NRF2 drives malignant progression of head and neck squamous cell carcinoma through glucose-6-phosphate dehydrogenase and transketolase activation

Ya-Chu Tang[1,2], Jenn-Ren Hsiao[3,4], Shih-Sheng Jiang[5], Jang-Yang Chang[2,5,6], Pei-Yi Chu[5,7], Ko-Jiunn Liu[5], Hsun-Lang Fang[8], Li-Mei Lin[2], Huang-Hui Chen[2], Yen-Wen Huang[5], Yu-Tsen Chen[5], Fang-Yu Tsai[5], Su-Fang Lin[5], Yung-Jen Chuang[9,10*], Ching-Chuan Kuo[2,11*]

[1] Graduate Program of Medical Biotechnology, National Tsing Hua University, Hsinchu, Taiwan

[2] Institute of Biotechnology and Pharmaceutical Research, National Health Research Institutes, Miaoli, Taiwan

[3] Department of Otolaryngology, National Cheng Kung University Hospital, College of Medicine, National Cheng Kung University, Tainan, Taiwan

[4] Institute of Clinical Medicine, College of Medicine, National Cheng Kung University, Tainan, Taiwan

[5] National Institute of Cancer Research, National Health Research Institutes, Miaoli, Taiwan

[6] Department of Internal Medicine, College of Medicine, National Cheng Kung University, Tainan, Taiwan

[7] Department of Pathology, Show Chwan Memorial Hospital, Changhua, Taiwan

[8] Department of Cosmetology and Health Care, Min-Hwei College of Health Care Management, Tainan, Taiwan

[9] Institute of Bioinformatics and Structural Biology, National Tsing Hua University, Hsinchu, Taiwan

[10] Department of Medical Science, National Tsing Hua University, Hsinchu, Taiwan

[11] Graduate Institute of Biomedical Sciences, China Medical University, Taichung, Taiwan

*Correspondence should be addressed to C.-C.K. (cckuo@nhri.org.tw) or Y.-J.C. (yjchuang@life.nthu.edu.tw)

SUPPLEMENTARY MATERIALS AND METHODS

Materials. All chemicals, apart from described in the methods, were obtained from E. Merck Co. (Darmstadt, Germany) or Sigma-Aldrich (St. Louis, MO) and were standard analytic grade or higher.

Cell lines. The carcinogen-transformed DOK and Ca9-22-D1 cells were established by Dr. Ching-Chuan Kuo's laboratory (Institute of Biotechnology and Pharmaceutical Research, National Health Research Institutes of Taiwan). In brief, the Ca9-22-D1 cell line was selected from a Ca9-22 xenograft tumor based on greater *in vivo* tumorigenic properties compared to parental Ca9-22 cells (**Figure S1**). DOK cells were treated with non-toxic concentration of carcinogens (nicotine: 500 μM; NNK: 10 μM; arecoline: 50 or 100 μM) for 12 months, and the carcinogen-transformed cells, including nicotine-, NNK-, and arecoline-tolerant DOK cells, were established (**Figure S2**). In this study, we used nicotine- and arecoline-transformed DOK cells for further investigation.

Pathway analysis. Total RNA was isolated from NRF2-knockdown HONE-1 and Ca9-22-D1 cells by RNeasy Midi Kit (Qiagen, Hilden, Germany), and subjected to microarray analysis using the Affymetrix Human Gene 2.0 ST array (Thermo Fisher Scientific, Waltham, MA, USA). Microarray data was further assessed by gene set enrichment analysis (GSEA). The gene sets with both nominal (NOM) *p*-value less than 0.05 and false discovery rate (FDR) q-value less than 0.25 were considered statistically significant.

Web-based data-mining analysis. (1) Survival analysis: level 3 TCGA data on HNSCC was obtained from the cBioPortal for Cancer Genomics (http://cbioportal. org). The survival analysis was performed using the Kaplan-Meier method and statistically significant differences in survival were determined using the log-rank test. Survival curve development and statistical analyses were performed using GraphPad Prism 5.0 Software (GraphPad Software Inc., San Diego, CA, USA). (2) Gene correlation analysis: the mRNA expression levels of NRF2, G6PD, and TKT in HNSCC were analyzed using the Oncomine (www.oncomine.org) database of cancer expression profiling data.

Measurement of intracellular redox status and G6PD/TKT enzyme activities. To study the effects of NRF2 knockdown on G6PD and TKT enzymatic activities, HNSCC cells were transfected with NRF2-siRNA or scrambled-siRNA and the whole cell homogenates were collected 48 h after transfection. For G6PD, 100 μg of whole cell

homogenate per sample was assessed using the Glucose-6-Phosphate Dehydrogenase Activity Colorimetric Assay Kit (BioVision Inc., Milpitas, CA, USA) according to the manufacturer instructions. The TKT enzyme activity assay was performed according to previously established methods[1-4] with slight modification. In brief, following transfection with NRF2-siRNA or non-targeted control siRNAs, HNSCC cells were lysed in 50 mM glycylglycine (pH 7.6) buffer and homogenized through ultrasonication on ice. After centrifugation at 14,000 g for 10 min at 4°C, supernatants were collected and total protein concentration was determined by Bradford assay. An aliquot of cell extract containing 100 µg of whole cell homogenate per sample was added to the reaction mixture, for a final volume of 150 µL, containing 50 mM glycylglycine (pH 7.6), 10 mM sodium arsenate, 0.4 mM NAD^+, 3.2 mM dithiothreitol, 2.5 mM $MgCl_2$, 0.2 mM thiamine diphosphate, 3 units of glyceraldehyde 3-phosphate dehydrogenase and 15 mM D-ribose 5-phosphatedisodium salt dehydrate. The absorbance variation of the mixture at 340 nm was measured by a Multiskan™ GO microplate spectrophotometer in kinetic mode for two hours. The TKT enzyme activity assay was fully validated in-house using the transient TKT-siRNA knockdown. The TKT enzymatic activity was then calculated as the difference in absorbance between two time points (T1 and T2) in the reaction linear range. The results were presented as a percentage of the control group.

RNA preparation and quantitative real-time polymerase chain reaction. Total RNA was isolated from the cultured cells using the Nucleospin RNA Kit (Macherey-Nagel, Düren, Germany) according to the manufacturer's instructions. After extraction, the RNA solution was quantified by NanoDrop spectrophotometry and stored at -80°C until use. To synthesize single-strand cDNA, an aliquot of 1 µg of total RNA from cultured cells was primed with 1 µL random hexamers (50 µM; Invitrogen, Carlsbad, CA, USA) in a final volume of 12 µL at 65°C for 5 min. After being chilled on ice for 1 min, the cDNA was synthesized at 50°C for 1 h with 4 µL of 5X first-strand buffer [250 mM Tris-HCl (pH 8.3), 375 mM KCl, 15 mM $MgCl_2$], 1 µL of dNTP mix (10 mM of each dNTP), 1 µL of DTT (100 mM), 1 µL (200 units/µL) SuperScript™ III Reverse Transcriptase (Invitrogen, Carlsbad, CA, USA), and 1 µL of recombinant ribonuclease inhibitor (40 units/µL; Invitrogen, Carlsbad, CA, USA). To inactive the reactions, the mixtures were heated at 70°C for 15 min and the cDNA products were stored at -20°C. To measure the level of NRF2 and PPP-related enzyme mRNA, we performed quantitative real-time RT-PCR analysis of three biological

replicates using the ViiA™ 7 Real-Time PCR System (Applied Biosystems, Foster City, CA, USA). The real-time PCR was performed in a 10 µL reaction mixture containing 1 µL of the 10-fold diluted cDNA template solution, 5 µL 2X qPCR master mix (Kapa Biosystems, Wilmington, MA, USA), and 0.3 µL of both forward and reverse primers (5 µM). The thermal cycling was performed under the following conditions: 3 min incubation at 95°C for initial denaturation, 40 cycles of PCR amplification at 95°C for 3 seconds and 60°C for 30 seconds. The Ct values of NRF2 and PPP-related genes were normalized to the amount of input cDNA calculated relative to levels of the housekeeping gene RPLP0. The relative quantity of the target gene was calculated using the $2^{-\Delta\Delta CT}$ formula and the data were presented as the mean percentage compared control. The real-time PCR primer pairs used for target gene amplification are listed in **Table S2**.

Western blot analysis. Preparation of cell lysate for western blot was performed as follows. First, cells were washed with ice-cold PBS, scraped from the Petri dish, and collected in a 1.5 mL Eppendorf tube. Next, the cell pellets were lysed in cold CelLytic M cell lysis reagent containing a final concentration of 1 mM PMSF, 1 mM DTT, 2 mM Na_3VO_4 and EDTA-free protease inhibitor cocktail (1 tablet/50 mL; Roche Diagnostics GmbH, Penzberg, Germany). After ultrasonic homogenization in an ice bath, the cell lysates were centrifuged at 14,000 g for 10 min at 4°C and the supernatants were transferred to the fresh tubes. We used the Bradford assay (Bio-Rad Laboratories, Hercules, CA, USA) to determine total protein concentration. An aliquot of extracted protein containing 40 µg of whole-cell lysate and 1× electrophoresis sample buffer (50 mM Tris-HCl pH 6.8, 10% glycerol, 2% SDS, 100 mM DTT, and 0.05% bromophenol blue) was then used in the western blot assay. To determine nuclear NRF2 protein levels in HNSCC cell lines, nuclear fractions were isolated according to the following protocol. Cells were washed in ice-cold PBS, cells were pelleted via centrifugation in a 1.5 mL microcentrifuge tube and gently re-suspended in 400 µL ice-cold Buffer A (10 mM Tris-HCl pH 7.9, 10 mM KCl, 0.1 mM EDTA, 0.1 mM EGTA, 1mM DTT, 1 mM PMSF, 1× protease inhibitor cocktail, and 1 mM NEM). After incubation on ice for 15 minutes, 25 µL 10% NP40 was added and mixed vigorously using a vortex at the highest setting for 10 seconds. The mixture was centrifuged at 14,000 g for 30 min at 4°C and the supernatant (cytoplasmic fraction) was transferred to a fresh 1.5 mL microcentrifuge tube. The resulting pellet was washed in 500 µL ice-cold Buffer A three times and then re-suspended in ice-cold

Buffer B (20 mM Tris-HCl pH 7.9, 40 mM NaCl, 1 mM EDTA, 1 mM EGTA, 1mM DTT, 1 mM PMSF, 1× protease inhibitor cocktail, and 1 mM NEM). The mixture was incubated on ice for 15 minutes with vigorously mixing via vortex at the highest setting for 10 seconds every 2-3 minutes. The mixture was then centrifuged for 10 min at 14,000 rpm at 4°C. The supernatant (nuclear fraction) was collected and total protein concentration was assessed by Bradford assay. The antibodies used to recognize target proteins are listed in **Table S3**.

Soft agar colony formation assay. To assess capacity for anchorage-independent growth, cells were cultured in 0.35% low-melting agarose (Invitrogen, Waltham, MA, USA) at 500 cells per 6-well plate. The cells were incubated in a humidified atmosphere containing 95% air/5% CO_2 at 37 °C. The complete growth medium on top of the agar was changed every three days. After15–20 days of culture, the colonies were stained with 0.5% (w/v) methylene blue in 95% ethanol for 30 minutes and all visible colonies were counted. The data on colony formation efficiency were reported as a percentage relative to the control group.

Trans-well migration and invasion assays. The trans-well migration and invasion assays were performed to evaluate cell motility and invasiveness, respectively. Briefly, 1×10^5cells in 300 µL serum-free culture medium were seeded in each chamber of 24-wellcell culture inserts with 8.0 µm pores (Corning Inc, Corning, NY, USA) for the migration assay and in Matrigel matrix-coated cell culture inserts (Corning Inc, Corning, NY, USA) for the invasion assay. The complete culture medium was added to each lower chamber to attract cells toward the basal compartment After 16–20 h of incubation at 37 °C, the cells that had migrated to or invaded the basal side of the cell culture insert were fixed in methanol for 30 min. Before staining the cells that migrated or invaded with 0.05% (w/v) crystal violet in 95% ethanol, we scraped off the cells that remained on the upper membrane using cotton swabs. Each culture insert was photographed under an IX71 Inverted Fluorescence Microscope (Olympus Corporation, Shinjuku, Tokyo, Japan) and was quantified using ImageJ. To study the effect of ROS on NRF2 knockdown-induced reduction of cell motility, NRF2-siRNA and scrambled-siRNA treated cells were pre-incubated with or without 10 mM NAC for 4h. After pretreatment with NAC, we assessed cell motility using the trans-well migration assay. To study the effects of glucose 6-phosphate, ribose 5-phoshate and ribulose 5-phosphate on NRF2 knockdown-induced reduction of cell

motility and invasiveness, D-glucose 6-phosphate monosodium salt, D-ribose 5-phosphate disodium salt dehydrate, or D-ribulose 5-phosphate disodium salt were added into the cell culture insert. After 16–20 h, the cells that had migrated or invaded were stained with 0.05% crystal violet and quantified via ImageJ.

Cell proliferation assay. To investigate the impact of NRF2 on cell growth, NRF2-siRNA and scrambled-siRNA treated cells were re-seeded at a density of 6 x 10^3 cells per well in 24-well plate. After 72 h of incubation at 37 °C, the cells were stained with 0.5% (w/v) methylene blue in 95% ethanol for 1h and washed in water. The cell-bound dye was dissolved in 1% N-Lauroyl sarcosine sodium. The absorbance of the N-Lauroyl sarcosine sodium solution was measured at 595 nm (A595) using a Multiskan™ GO microplate spectrophotometer (Thermo Fisher Scientific, Waltham, MA, USA). The growth index was determined using the following formula: [(A595 treated cells 72 h -A595 treated cells 16h)/ (A595 control 72 h -A595 control 16h)] × 100%. To study the effect of NAC on NRF2 knockdown-induced reduction of cell growth, NRF2-siRNA and scrambled-siRNA treated cells were re-plated at a density of 6 x 10^3 cells per well in 24-well plates. After overnight incubation at 37 °C, the cells were treated with or without the indicated concentrations of NAC for 72 h and the cell number was determined by methylene blue staining.

Intracellular ROS and GSH detection. Intracellular ROS level was evaluated using the dichloro-dihydro-fluorescein diacetate (DCFH-DA) assay. Cells were plated at a density of 6 x 10^5 into 6 cm culture dishes with complete culture medium. After overnight incubation at 37 °C, the cells were washed with pre-warmed PBS, and harvested by trypsinization. Complete culture medium (without Phenol Red) was added to stop trypsinization and cells were re-suspended. Cells were collected in 1.5 mL microcentrifuge tubes and centrifuged for 5 min at 200 x g at room temperature; the supernatant was aspirated. The cell pellet was re-suspended in serum-free medium (without Phenol Red) with 25 µM 2',7'-dichlorofluorescin diacetate by gently pipetting up and down (500 µL/tube). Suspended cells were placed in a cell incubator [(37 °C), high relative humidity (95%), and controlled CO_2 level (5%)] in the dark for 30 min. The cells were then centrifuged for 5 min at 200 x g at room temperature and the supernatant was aspirated. The cells were then washed 3 times with serum free medium (without phenol red). Cells were re-suspended cells in complete culture medium (without Phenol Red) by gently pipetting up and down (500 µl/tube). The cells were immediately subjected to fluorescence intensity detection via flow

cytometry. A total of 10,000 events from each group were analyzed by flow cytometry (FACSCalibur, BD Biosciences, Franklin Lakes, NJ, USA). To study the effect of NAC on NRF2 knockdown-stimulated upregulation of oxidative stress, NRF2-siRNA and scrambled-siRNA treated cells were pre-treated with or without 10 mM NAC for 4 h and the intracellular ROS level was measured using the DCFH-DA assay, as previously described. The intracellular GSH content was measured using a thiol-reactive dye, monochlorobimane. Cells were plated at a density of 1 x 10^4 per well in 96-well plate with complete culture medium. After overnight incubation at 37 °C, the cells were stained with 100 μM monochlorobimane at 37 °C for 1 h and the fluorescence intensity at 390/520 nm (excitation/emission) was detected using the FlexStation 3 multi-mode microplate reader (Molecular Devices, San Jose, CA, USA).

Metabolite analysis. The measurement and quantification of metabolic intermediates were employed by Human Metabolome Technologies Inc. (Tsuruoka, Japan). For CE-TOFMS analysis, Ca9-22-D1 cells were transfected with NRF2-siRNA or scrambled siRNA for 48h. To assess whether the R5P supply can effectively restore NRF2-knockdown induced reduction in intracellular R5P, transient NRF2-knockdown Ca9-22-D1 cells were treated 240 μM R5P for 1 and 20 h. After R5P incubation, UPLC-ESI-MS/MS method was used for the measurement and quantification of intracellular R5P. The intracellular extracts were obtained from 2 × 10^6 cells per sample by using methanol according to the instructions for use.

In vivo experiments. All of the experimental procedures were approved by the Institutional Animal Care and Use Committee (IACUC) of NHRI, Taiwan and were performed according to the *Guide for the Care and Use of Laboratory Animals* (8th edition. Washington, DC: National Academies Press US; 2011). To study the effects of NRF2 on the metastatic potential of HNSCC cell lines, stable NRF2-knockdown HONE-1 and Ca9-22-D1 cells were used in the experimental lung metastasis assay according as follows. First, the cells were trypsinized at 70%–80% confluence, washed twice with HBSS and counted with a hemocytometer. Next, 2x10^5 cells were suspended in 100 μL HBSS and injected intravenously into 6- to 8-week-old NOD/SCID mice using a 1 mL syringe with a 27G x 1/2” needle. Mice were sacrificed after 25 (Ca9-22-D1 cells) or 50 (HONE-1 cells) days. The lungs were harvested and fixed in Bouin’s solution at 4°C for 24–48 hours. Before paraffin embedding, the number of the metastatic tumor foci on the lung surfaces within each mouse was counted. For the *in vivo* tumor growth assay, the cells were harvested at approximately 70–80%

confluence, re-suspended in HBSS, and mixed with an equal volume of Matrigel to achieve a final concentration of 5 × 10^6 cells/ 100 µL (DOK cells) or 3 × 10^6 cells/ 100 µL (Ca9-22-D1 cells). The cell-Matrigel mixture (100 µL per mouse) was injected subcutaneously and then tumor volume and body weight were recorded once per week (DOK cells) or twice per week (Ca9-22-D1 cells). For histologic analysis, the formalin-fixed tissues were embedded in paraffin, sliced at a thickness of 4 µM, and stained with hematoxylin and eosin (H&E).

Immunohistochemistry. After fixation in 10% buffered formalin or Bouin's solution at 4°C for 24–48 h, the tissues were dehydrated and embedded in paraffin wax. The paraffin-embedded tissues were dissected into slices of 4 µm thickness and deparaffinized in three changes of fresh xylene for 5 minutes each. Following deparaffinization, the tissue sections were rehydrated in a graded series of ethanol and immersed twice in phosphate-buffered saline (PBS) for 5 min. To suppress the activity of endogenous peroxidase, the tissue sections were incubated with hydrogen peroxide. Sections were then heated with 10 mM citrate buffer (pH 6) for 15 min at 97°C to induce antigen retrieval. After cooling to room temperature, the tissue sections were washed in two changes of PBS for 5 minutes each. Prior to primary antibody incubation at 4˚C overnight, the tissue sections were pre-blocked with serum blocking reagent (4% goat serum in PBS) at room temperature for 30 min. Immuno-reactive signals were detected via N-Histofine® Simple Stain Mouse MAX PO (Nichirei Bioscience, Tokyo, Japan), visualized using the DAB Peroxidase (HRP) Substrate Kit (Vector Laboratories, Burlingame, CA, USA) and sections were counter staining with GM Hematoxylin Stain solution (Muto Pure Chemical Co., Ltd., Tokyo, Japan). The primary antibody against NRF2 is listed in **Table S3.**

SUPPLEMENTARY FIGURES

Figure S1

A

Locus	Test sample		Reference
	Ca9-22-D1	Ca9-22	Ca9-22 (JCRB0625)
D7S820	11, 13	11, 13	11, 13
CSF1PO	11, 12	12, 12	12, 12
THO1	6, 6	6, 6	6, 6
D13S317	11, 12	11, 11	11, 11
D16S539	9, 10	9, 10	9, 10
vWA	16, 17	16, 16	16, 16
TPOX	8, 11	8, 11	8, 11
Amelogenin	X, Y	X, Y	X, Y
D5S818	11, 12	12, 12	12, 12
Percent Match	86.7%	100%	-

B

Gene	Ca9-22-D1		Reference “CCLE Ca9-22”
	Protein Change	Type	
ABL2	p.R285*	Stop_gained	p.R285*
TP53	p.R248W	Missense	p.R248W
CTNNA1	p.E865*	Stop_gained	p.E865*
CYP2C19	p.S18P	Missense	p.S18P
DICER1	p.S678F	Missense	p.S678F
ERBB4	p.P854L	Missense	p.P854L
NCOA1	p.Q95K	Missense	p.Q95K
NFKB2	p.R199G	Missense	p.R199G
PIK3CG	p.E14*	Stop_gained	p.E14*
SAMD9	p.I268T	Missense	p.I268T

Figure S1 (continued)

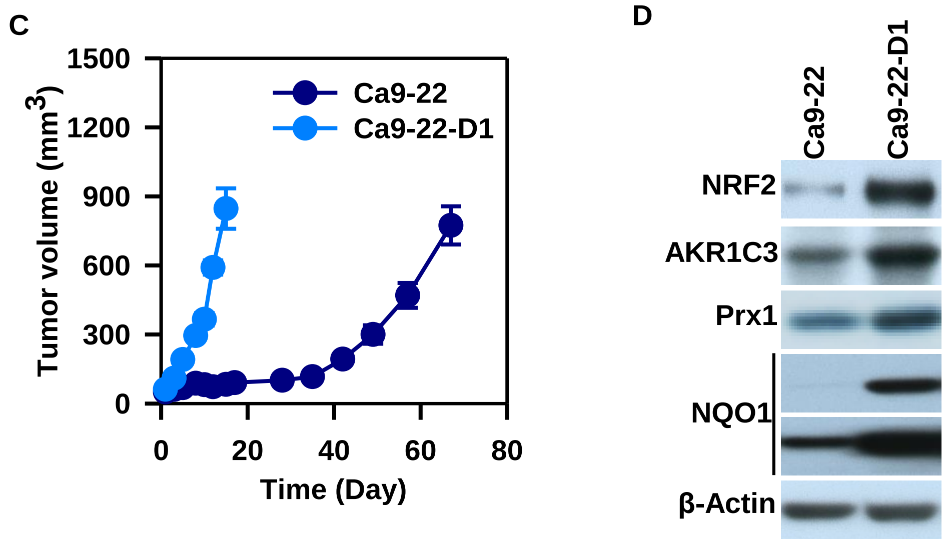

Figure S1. Characterization of Ca9-22-D1 cell line.

The Ca9-22-D1 cell line is 86.7% genetically similar to the Ca9-22 HNSCC cell line and possesses more malignant characteristics. Comparison between Ca9-22 and Ca9-22-D1 cells using short tandem repeat (STR) DNA profiles (A) and next-generation sequencing (NGS). The Short Tandem Repeat (STR) DNA profiling of Ca9-22 and Ca9-22-D1 cell lines was carried out by MISSION BIOTECH CO., LTD. (Taipei, Taiwan) and Food Industry Research and Development Institute (Hsinchu, Taiwan), respectively. The next-generation sequencing (NGS) analysis of Ca9-22-D1 cell line was carried out by ACT Genomics Co. LTD. (Taipei, Taiwan). (C) Tumor growth curves depict the tumor volume of Ca9-22 (dark blue) versus Ca9-22-D1 (light blue). (D) Protein levels of NRF2 and NRF2-downstream targets, AKR1C3, Prx1 and NQO1, in Ca9-22 and Ca9-22-D1 cell lines.

Figure S2

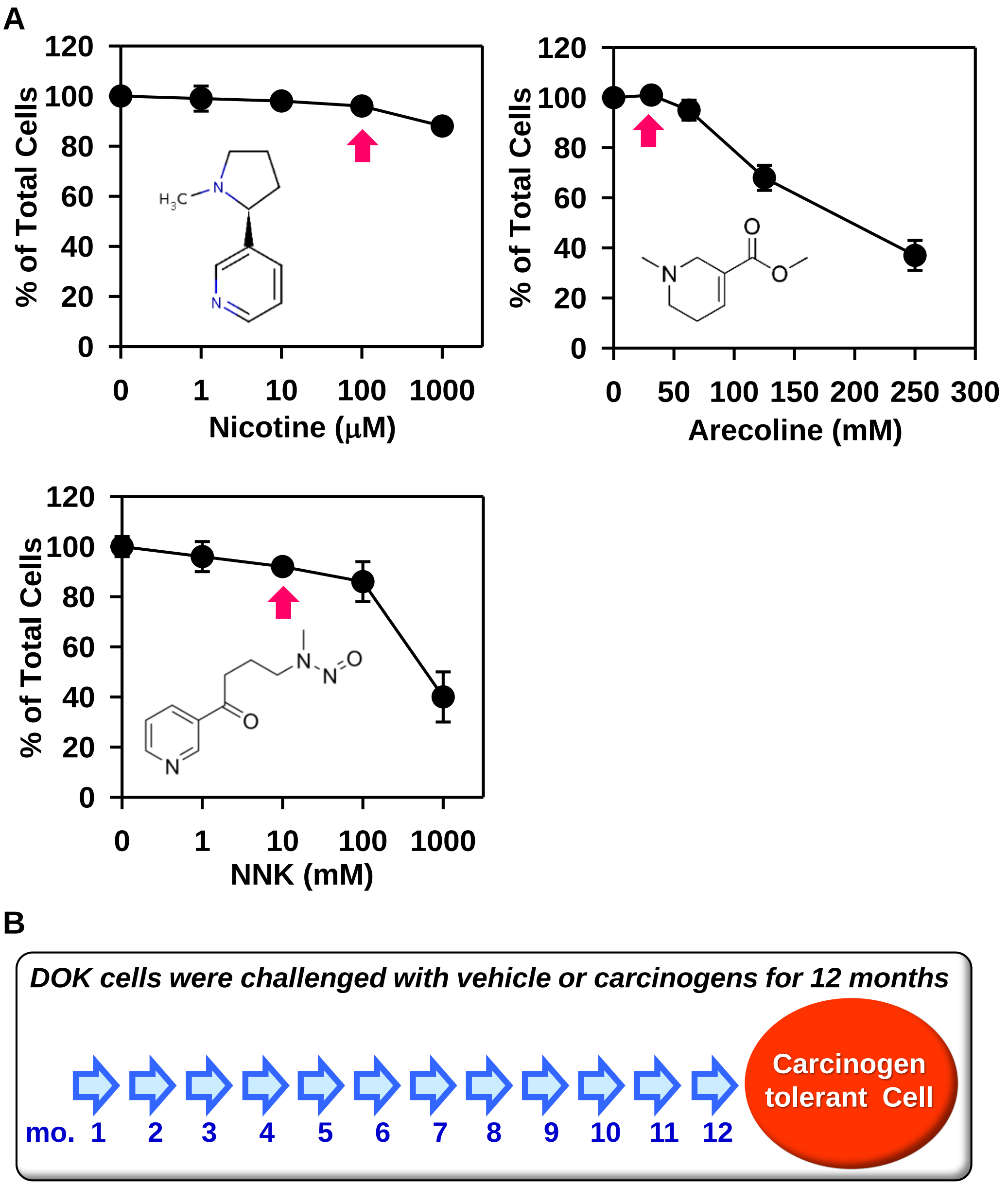

Figure S2. Establishment of carcinogen-transformed DOK cells.

(A) Oral dysplasia DOK cells were treated with different concentrations of nicotine, NNK, or arecoline for 3 generations, and maximum non-toxic concentrations of these carcinogens were determined. (B) The flow chart for establishment of carcinogen-transformed DOK cells. DOK cells were treated with vehicle or non-toxic concentrations of carcinogens (nicotine: 500 μM; NNK: 10 μM; arecoline: 50 and 100 μM) for 12 months, and the carcinogen-transformed cells, including nicotine-, NNK-, and arecoline-tolerant DOK cells, were established for use in subsequent studies.

Figure S3

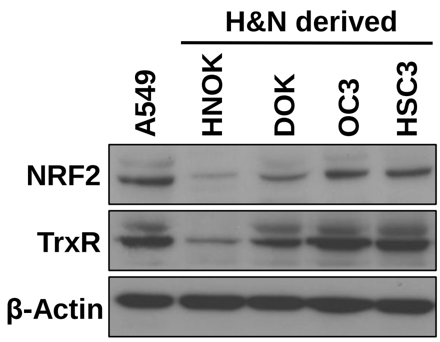

Figure S3. NRF2 is correlated with malignant features in human head and neck-derived cells.

The expression level of NRF2 protein was determined in normal human oral keratinocytes (HNOK), dysplasia oral keratinocytes (DOK), and two human HNSCC cell lines, OC3 and HSC3. A549, a lung cancer cell line in which NRF2 was constitutively stabilized due to a somatic mutation in the KEAP1 gene, was used as a positive control.

Figure S4

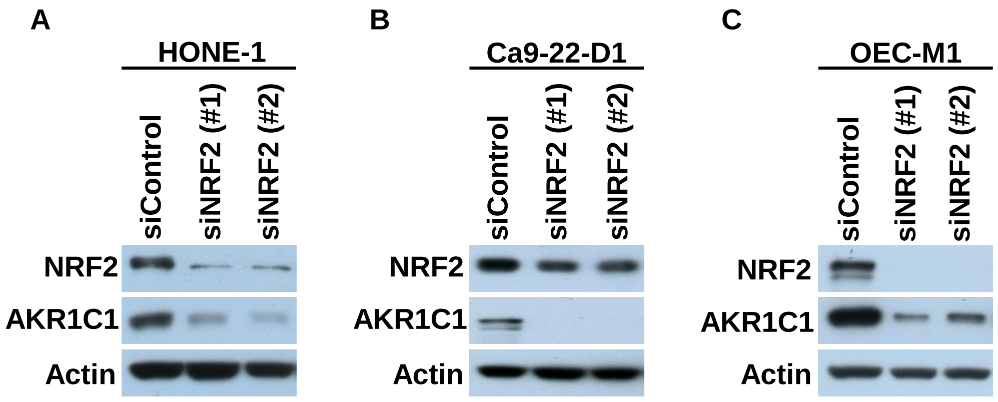

Figure. S4. NRF2 protein levels in NRF2-knockdown HNSCC cells.

The knockdown efficiencies of NRF2-siRNA were validated by assessing expression of NRF2 and AKR1C1, a downstream target of NRF2, in HONE1 (A), Ca9-22-D1 (B) and OEC-M1 (C) cells by Western blot analysis.

Figure S5

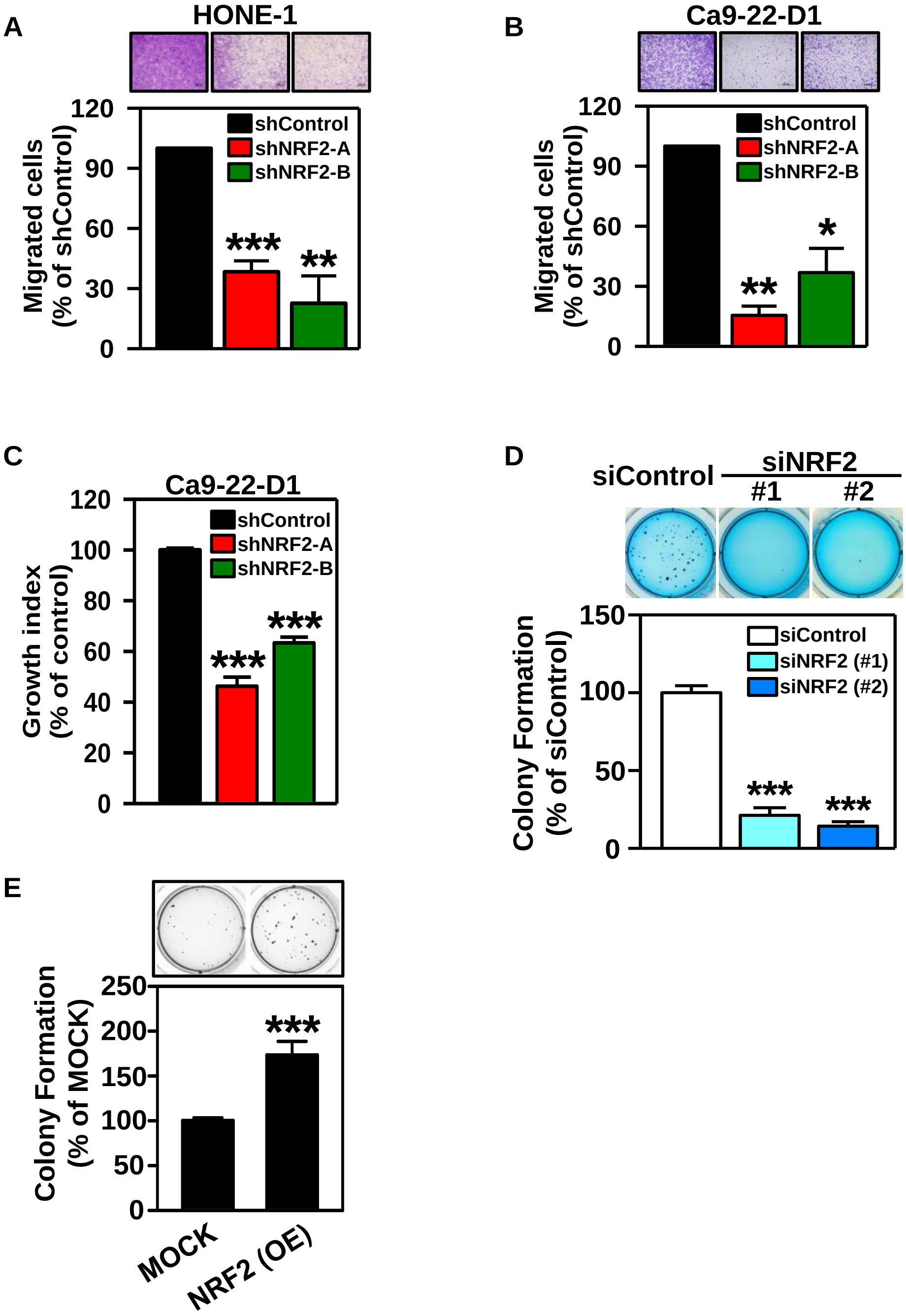

Figure S5. NRF2 promotes malignancy of HNSCC cells.

The migratory abilities of stable NRF2-knockdown Ca9-22-D1 (A) and HONE-1 (B) cells were evaluated by trans-well migration assay. (C) Effect of stable knockdown of NRF2 on cell growth in Ca9-22-D1 cells. Anchorage-independent growth of transiently NRF2-knockdown (D) and NRF2-overexpressing (E) HONE-1 cells were evaluated by soft agar colony formation assay. All data are expressed as the mean ± S.E. from three individual experiments. * $p < 0.05$; ** $p < 0.01$; *** $p < 0.001$. vs. shControl (A, B, C), siControl (D) or MOCK (E).

Figure S6

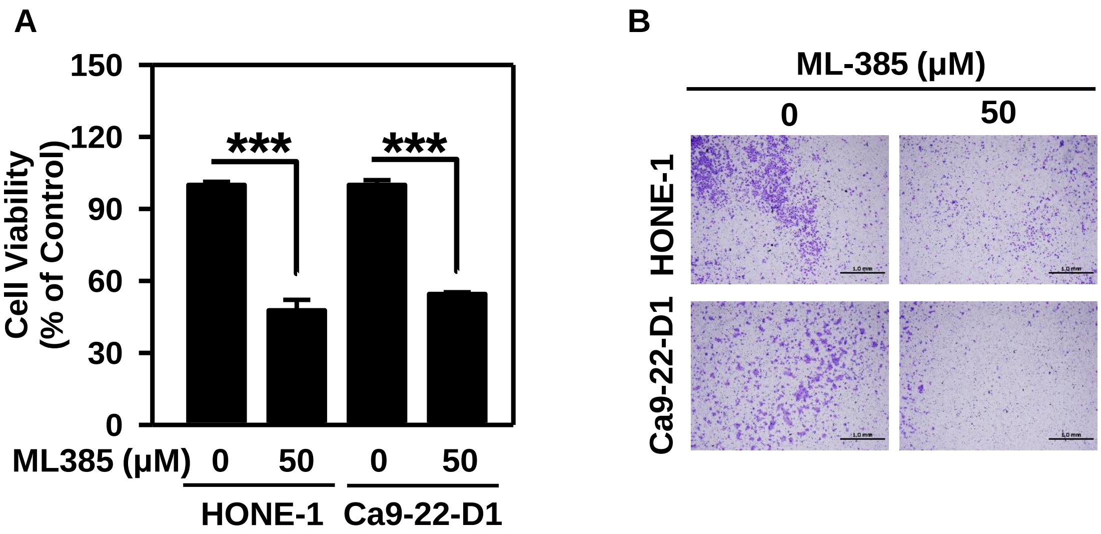

Figure S6. NRF2 inhibitor, ML-385, reduces cell viability and migration in HNSCC cells.

The effects of NRF2 inhibitor, ML-385, on cell viability (A) and migration (B) in HNSCC cells. All data are expressed as the mean ± S.E. from three individual experiments. * $p < 0.05$; ** $p < 0.01$; *** $p < 0.001$. vs. Control.

Figure S7

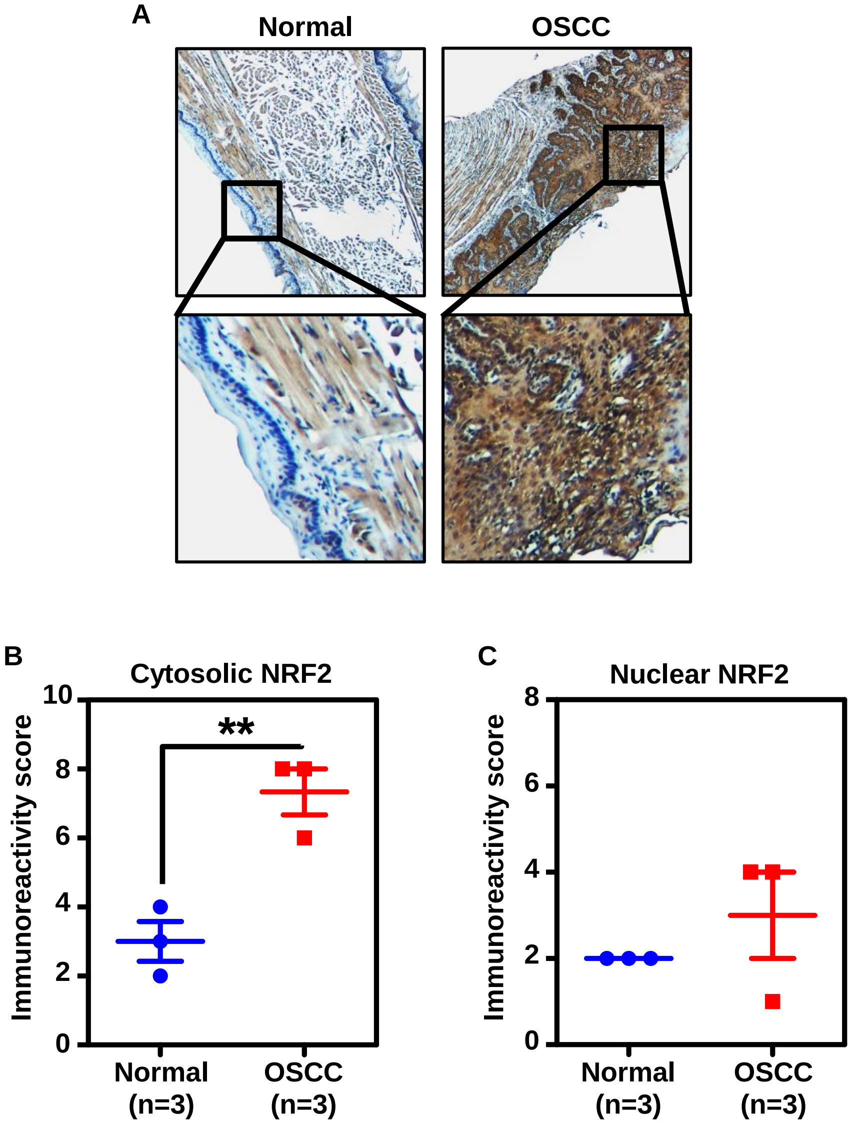

Figure S7. NRF2 protein level was up-regulated in carcinogen-induced mouse OSCC model.

The carcinogen-induced mouse OSCC model was developed through co-treating with arecoline and 4-nitroquinoline 1-oxide to mimic the effects of chronic betel quid chewing and tobacco consumption, respectively. The mice were sacrificed at the time when the occurrence of neoplasm in the tongue. The tongue was collected and processed for immunohistochemical staining. (A) Immunohistochemistry for NRF2 was performed on the tongue tissues of a mouse OSCC model. Quantitative results of cytosolic (B) and nuclear (C) NRF2 expression levels were estimated using the immunoreactivity scoring system (IRS). The IRS was calculated by multiplying the intensity of NRF2 staining (scale, 0–2) by the percentage of positive cells (4, > 80%; 3, 51–80%; 2, 10–50%; 1, < 10%; 0, 0%), which resulted in values ranging from 0 to 8. * $p < 0.05$; ** $p < 0.01$; *** $p < 0.001$. vs. Normal.

Figure S8

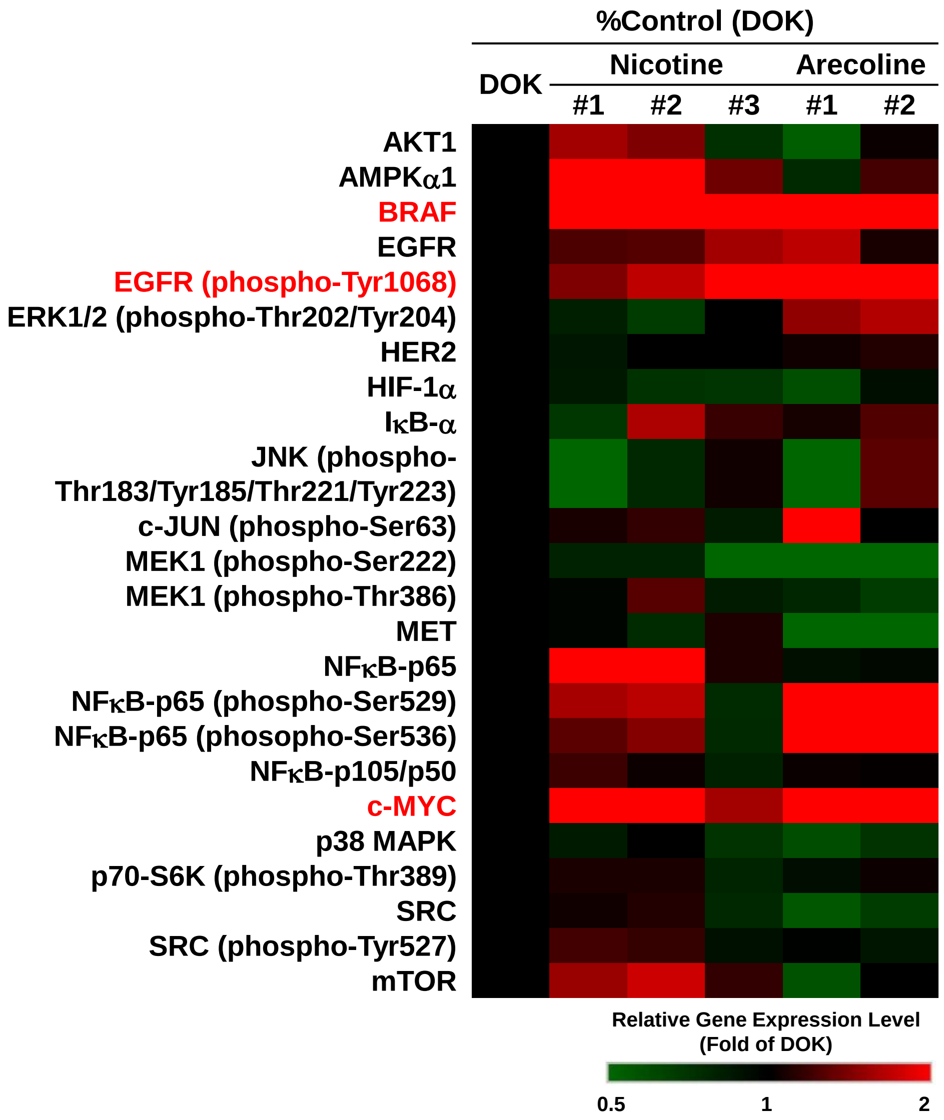

Figure S8. Changes in oncogene and signal transduction protein expression in carcinogen-transformed DOK cells.

Micro-Western Array was performed to analyze the levels of oncogenes and phosphorylation of signal-transduction proteins. The fold changes for particular proteins in carcinogen-transformed DOK cells was calculated relative to that in parental DOK cells. The results of the Micro-Western Array are illustrated heat-map. The net fold-change is color coded as indicated in the legend.

Figure S9

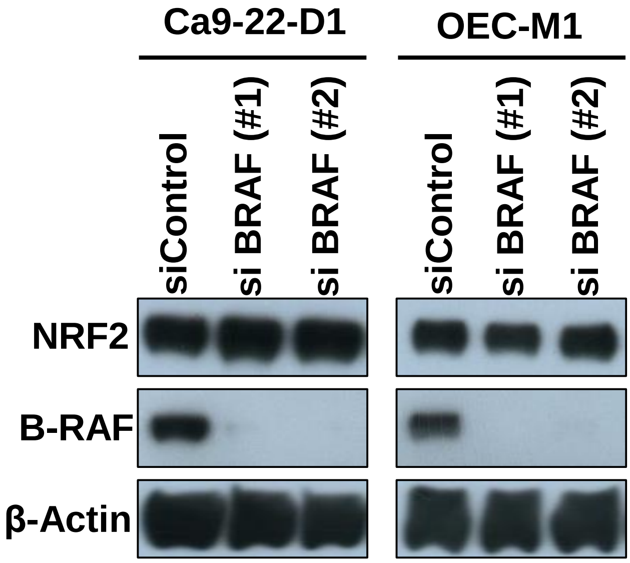

Fig. S9. Knockdown of BRAF do not alter NRF2 protein level in HNSCC cells.

The NRF2 expression in BRAF-knockdown HNSCC cells were examined by western blot.

Figure S10

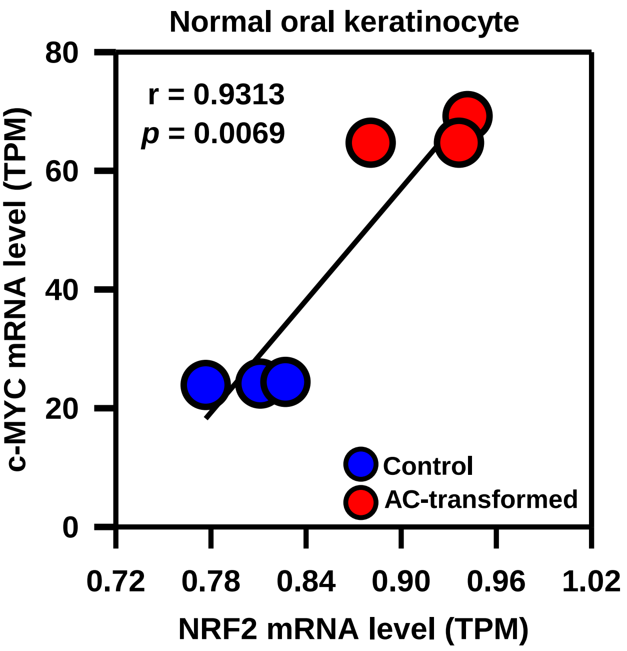

Fig. S10. The increases of both NRF2 and c-MYC expression were observed in arecoline (AC)-transformed normal oral keratinocyte cells.

The arecoline (AC)-transformed normal oral keratinocyte cells were established by chronic treatment of immortalized normal human oral keratinocyte (NHOK), OKF4/TERT-1 cells[5], with sub-lethal dosage of arecoline (12 μg/mL) for 8 months (unpublished data). For analysis of gene expression patterns, the total RNAs of paired control and AC groups from different clones, were collected and analyzed by mRNA sequencing. The NRF2 (X-axis) and c-MYC (Y-axis) mRNA levels were presented as TPM (Transcripts Per Kilobase Million) values. Blue dots: control group; red dots: arecoline-transformed group. Pearson's correlation coefficient (r) and *p*-values are shown for the analysis.

Figure S11

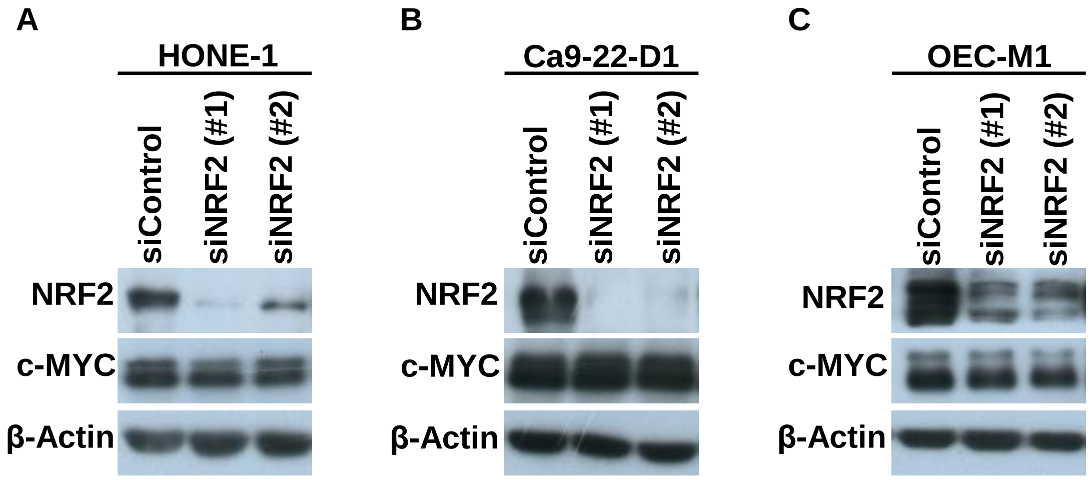

Fig. S11. The effects of NRF2 knockdown on c-MYC expression in HNSCC cells.
c-MYC protein levels were detected by western blot assay in NRF2-knockdown HONE-1 (A), Ca9-22-D1 (B) and OEC-M1 (C) cells after transfected with NRF2-siRNA or control-siRNA for 48 h.

Figure S12

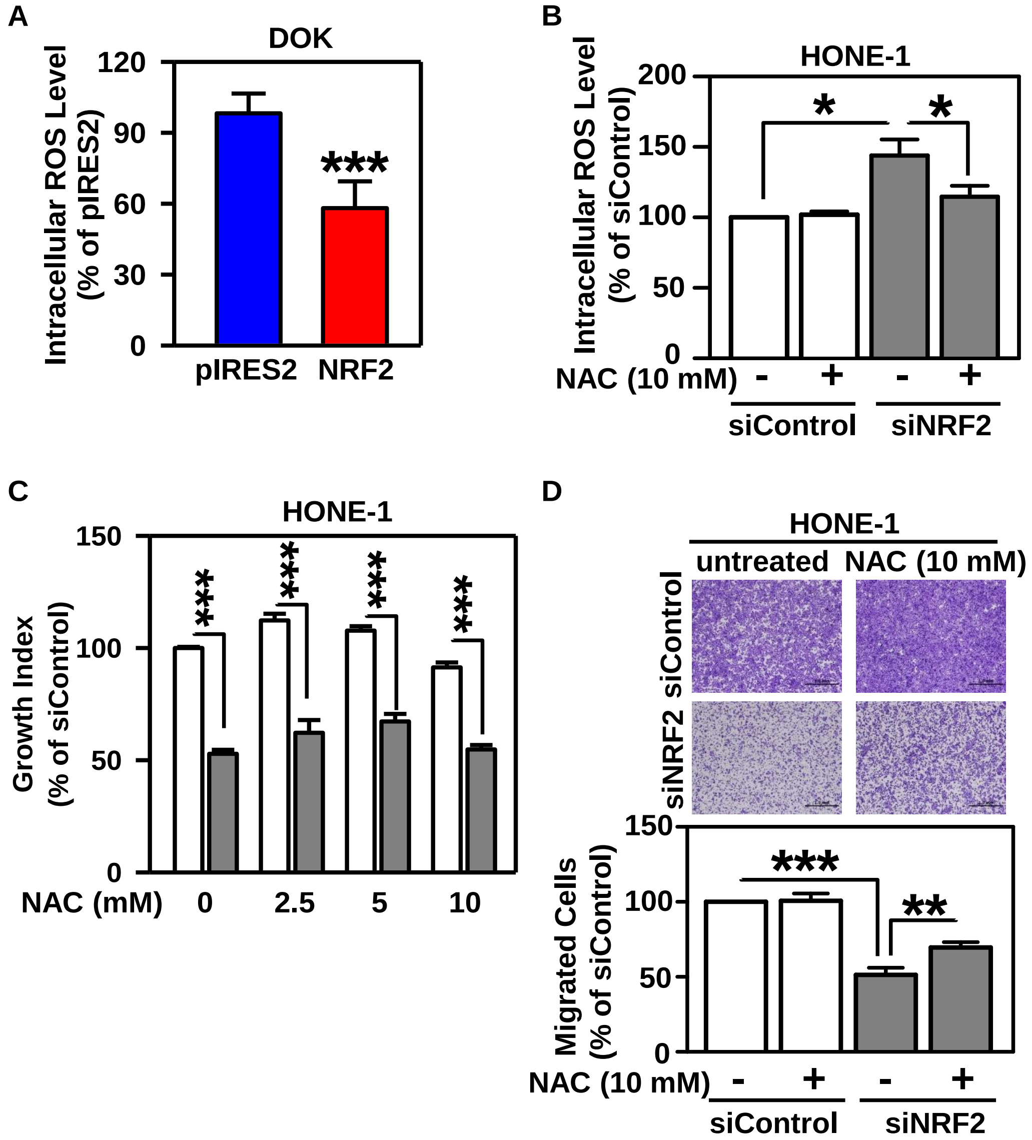

Figure S12. Redox status is marginally involved in NRF2-mediated HNSCC malignancy *in vitro*.

(A) Decreased intracellular ROS level in DOK cells stably over-expressing NRF2. (B) Effect of N-Acetylcysteine (NAC) on intracellular ROS levels in NRF2 knockdown HONE-1 cells. The relative intracellular ROS levels were normalized to that of the respective siControl group without NAC treatment. (C) The effect of elimination of ROS by NAC on cell growth in NRF2-knockdown HONE-1 cells. (D) NRF2-knockdown HONE-1 cells were assayed for migration ability in the absence and the presence of 10 mM NAC. The relative percentage of migrated cells was normalized to the siControl group without NAC treatment. * $p < 0.05$; ** $p < 0.01$; *** $p < 0.001$.

Figure S13

A

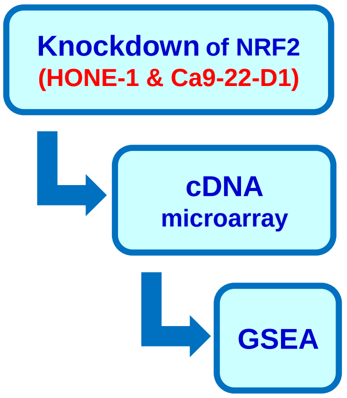

Top 30 down-regulated pathway in NRF2-kncokdown HNSCC cells	
**Pathway**	**Counts**
Cellular metabolism	10
Immune-related pathway	9
Cell growth and death signaling	7
Protein secretion and degradation	2
DNA repair	1
Prion diseases	1

B

Cellular metabolic pathways down-regulated in NRF2-knockdown cells (HONE-1 and Ca9-22-D1)

Gene sets	ES	NES	NOM p-val	FDR q-val
KEGG_PENTOSE_PHOSPHATE_PATHWAY	-0.584	-1.824	0.00391	0.01548
KEGG_VALINE_LEUCINE_AND_ISOLEUCINE_DEGRADATION	-0.511	-1.756	0.00403	0.02509
KEGG_PYRIMIDINE_METABOLISM	-0.419	-1.685	0.00381	0.03340
KEGG_GLYCOLYSIS_GLUCONEOGENESIS	-0.394	-1.451	0.01207	0.12246
KEGG_PURINE_METABOLISM	-0.325	-1.379	0.02633	0.23046
HALLMARK_OXIDATIVE_PHOSPHORYLATION	-0.366	-1.644	0.00000	0.00804
HALLMARK_XENOBIOTIC_METABOLISM	-0.326	-1.463	0.00595	0.03794
HALLMARK_HEME_METABOLISM	-0.326	-1.457	0.00204	0.03610
HALLMARK_FATTY_ACID_METABOLISM	-0.317	-1.371	0.02092	0.06943
HALLMARK_ADIPOGENESIS	-0.313	-1.406	0.00409	0.05582

Figure S13. NRF2 modulates HNSCC cell metabolism.
(A) A Flow diagram illustrating the analysis of pathways affected in NRF2-knockdown HNSCC cells (left). Number of pathways in Hallmarker and KEGG gene sets that are significantly downregulated in HONE-1 and Ca9-22-D1 cells via GSEA analysis, at a threshold of NOM p-value < 0.05 and FDR q-value < 0.25 (right). (B) Significantly down-regulated metabolic pathways identified by GSEA based on cDNA microarray analysis of gene expression in NRF2-knowckdown HNSCC cells.

Figure S14

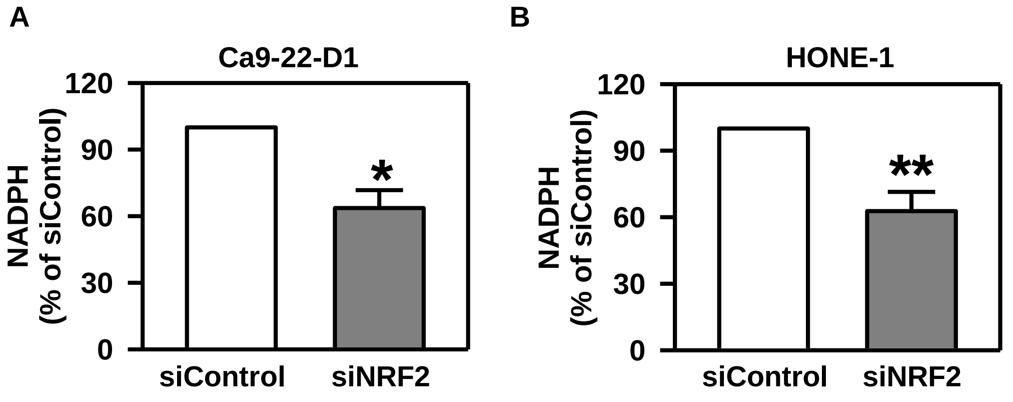

Figure S14. Knockdown of NRF2 reduces intracellular NADPH level.

Intracellular NADPH levels in Ca9-22-D1 (A) and HONE-1 (B) cells after transfection with NRF2-siRNA or non-targeted negative control. The relative intracellular NADPH levels were normalized to those of the siControl groups. All data are expressed as the mean ± S.E. from three individual experiments. * $p < 0.05$; ** $p < 0.01$; *** $p < 0.001$. vs. siControl.

Figure S15

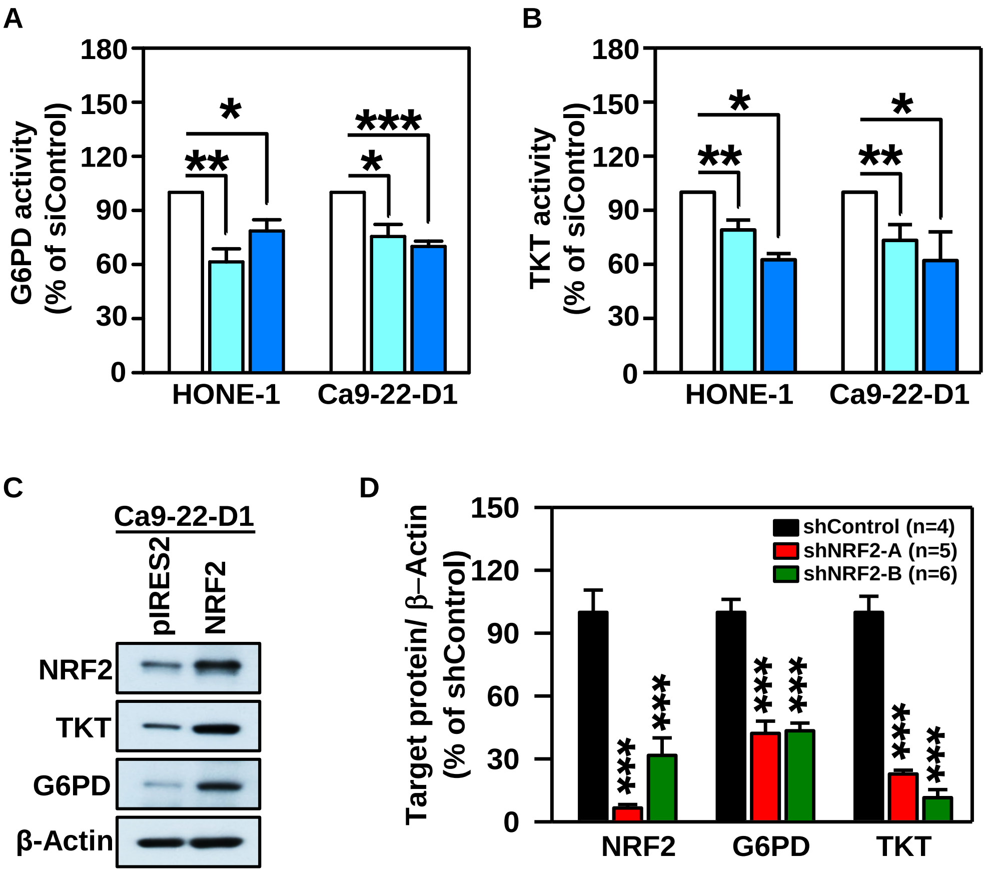

Figure S15. NRF2 regulates the pentose phosphate pathway in HNSCC cells. Measurements of G6PD (A) and TKT (B) enzyme activities in NRF2-knockdown HNSCC cells. (C) Total protein levels of NRF2, G6PD and TKT were assessed by western blot analysis of Ca9-22-D1 cells transiently overexpressing NRF2. β-Actin was detected as a loading control. (D) Expressions of NRF2, G6PD and TKT was measured by western blot assay of excised tumors from xenograft mice. The western blot images were analyzed using the Gel-Pro Analyzer and presented as the percentage of shControl after normalization to β-Actin. * $p < 0.05$; ** $p < 0.01$; *** $p < 0.001$

Figure S16

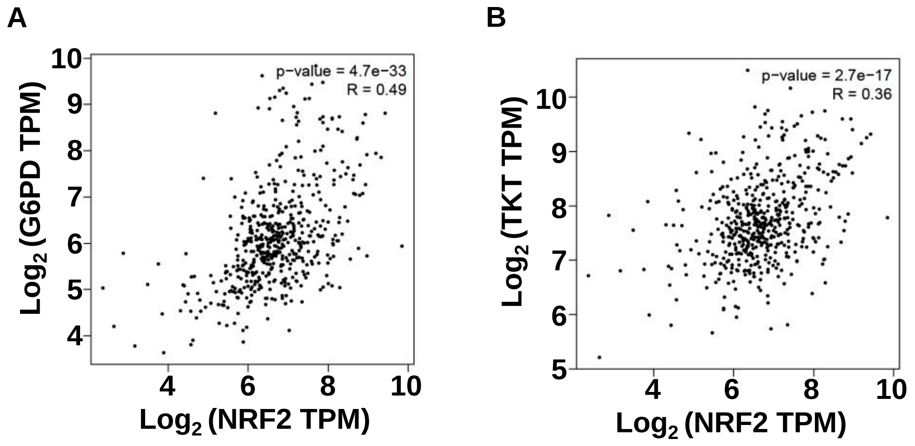

Figure S16. NRF2 level is positively associated with G6PD and TKT in patients with head and neck squamous cell carcinoma.

NRF2 expression was positively correlated with G6PD (A) and TKT (B) in the Cancer Genome Atlas (TCGA) HNSC dataset (Spearman's rank correlation coefficient, R = 0.49 and R = 0.36 respectively, $p < 0.001$).

Figure S17

A

Cisplatin combined with	Cell lines	Combination Index (CI)
DHEA	HONE-1	0.88
	OEC-M1	0.81
trans-polydatin	Ca9-22-D1	0.77
	OEC-M1	0.87
6-aminonicotinamide	HONE-1	0.07
	Ca9-22-D1	0.36

Figure S17. Synergistic cytotoxic effect of G6PD inhibitors and cisplatin in HNSCC cells.

(A) Cytotoxic effects on HNSCC cells co-treated with G6PD inhibitors and cisplatin for 72h. Combination index (CI) values of the combination therapy of Cisplatin and G6PD inhibitors were indicated respectively. The synergistic effect was demonstrated by a CI < 1 across a wide range of concentrations.

Figure S18

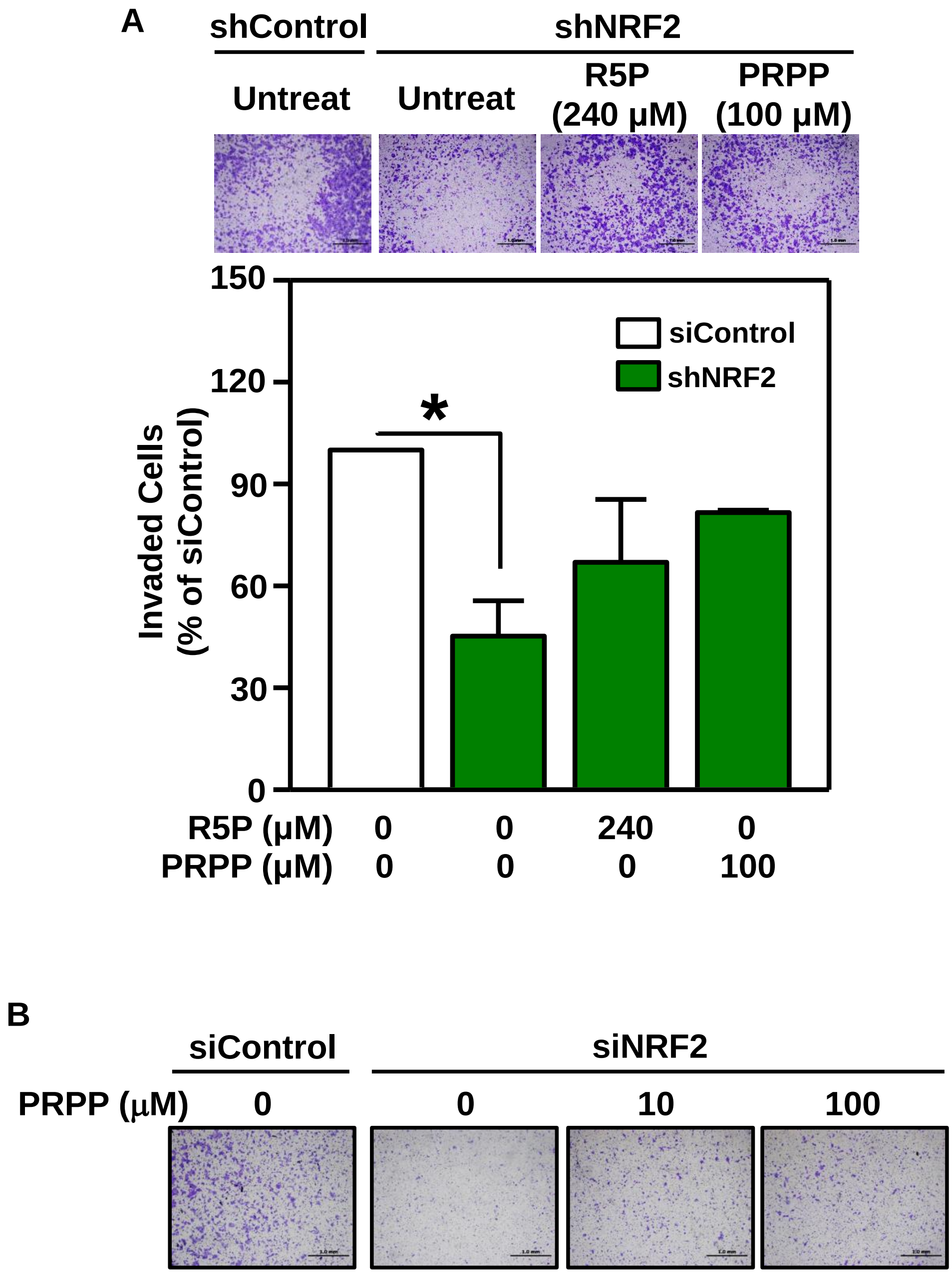

Figure S18. Decreased invasion ability by knockdown of NRF2 was restored by treatment with R5P and PRPP in HNSCC cells.

(A) Stable NRF2-knockdown Ca9-22-D1 cells were assayed for invasion ability in the absence and the presence of 240 μM R5P or 100 μM PRPP. The relative percentage of invaded cells was normalized to the shControl group without treatment. (B) Motility of transient NRF2-knockdown Ca9-22-D1 cells treated with or without PRPP was assessed using the trans-well migration assay. The relative percentage of invaded cells was normalized to the shControl group without treatment. * $p < 0.05$; ** $p < 0.01$; *** $p < 0.001$.

Figure S19

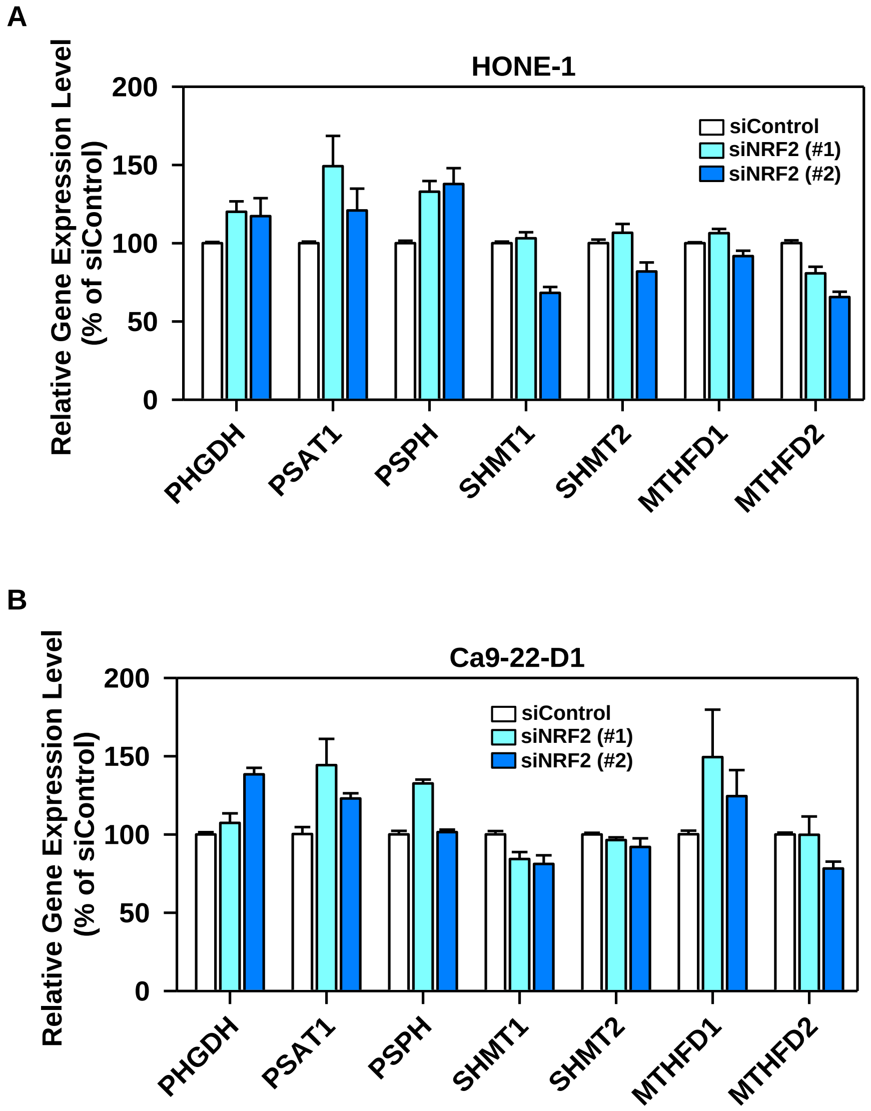

Figure S19. Knockdown of NRF2 did not alter the mRNA levels of enzymes involved in serine-glycine biosynthesis and nucleotide biosynthesis pathways in HNSCC.

The relative mRNA levels of enzymes involved in serine-glycine biosynthesis and nucleotide biosynthesis pathways in NRF2-knockdown HONE-1 (A) and Ca9-22-D1 (B) cells were validated by real-time PCR.

SUPPLEMENTARY TABLES

Table S1. Primers used for gene cloning

Name	Sequence
NRF2_Nhe1_F	GCTAGCaaaatgatggacttg
Nrf2_Xho1_R	CTCGAGaaactagtttttctt

Table S2. Primers used for quantitative real-time RT-PCR

Gene		Sequence	Reference (PMID)	Accession no.	Product size (bp)
hG6PD	FP	TGACCTGGCCAAGAAGAAGA	26583321	NM_000402.4	185
	RP	CAAAGAAGTCCTCCAGCTTG			
hPGLS	FP	GTCTCGATGCTAGCCCGC		NM_012088.2	163
	RP	TCGGCAGTCTGGAGAGAAGA			
hPGD	FP	TCTTCGGTTCTGCTCTGTCC		NM_002631.3	175
	RP	GTTCCCTTTGCCTCATTGGC			
hRPIA	FP	AATCTCATCAAGGGTGGCGG	26248089	NM_144563.2	155
	RP	GCCATTGGGATGACCTCGAT			
hRPE	FP	CACGGCCTTGGAAGTGAGAAA		NM_001278289.1	183
	RP	GGCTCCTTATGGTTTCACCGA			
hTKT	FP	GAAGATCAGCTCCGACTTGG	26811478	NM_001064.3	127
	RP	GTCGAAGTATTTGCCGGTGT			
hTALDO1	FP	TGACCCTCATCTCCCCATTT	22816071	NM_006755.1	265
	RP	ACCAGCTTGGCGTTGTCCTG			

Table S3. Antibody list

Antibody	Assay	Information
AKR1C1	Immunoblot	H00D1645A01 (Abnova)
β-Actin	Immunoblot	GTX109639 (GeneTex)
B-RAF	Immunoblot	SC-5284 (SantaCruz)
c-MYC	Immunoblot/ ChIP	ab32072 (Abcam)
EGFR	Immunoblot	sc-373746 (SantaCruz)
phospho-EGFR (Y1068)	Immunoblot	2234L (Cell Signaling Technology)
G6PD	Immunoblot	8866S (Cell Signaling Technology)
KEAP1	Immunoblot	MAB3024 (R&D)
Lamin B1	Immunoblot	ab20396 (Abcam)
NQO1	Immunoblot	3187S (Cell Signaling Technology)
NRF2	Immunoblot/ ChIP	ab62352 (Abcam)
NRF2	IHC staining	ab31163 (Abcam)
PGD	Immunoblot	13389S (Cell Signaling Technology)
TALDO1	Immunoblot	ab67467 (Abcam)
TKT	Immunoblot	8616S (Cell Signaling Technology)

Table S4. Primers used for ChIP-*q*PCR

Name		Sequence	Reference (PMID)
ChIP-hG6PD	FP	CTTTGGGGGAGTGCCAACAT	22789539
	RP	ATCACAAGGGCCATGGGCTT	
ChIP-hTKT	FP	GAAGAGGCTGGGACAGCTA	22789539
	RP	AAGAGCAGTATGAGGCAGGAGA	
ChIP-hNRF2	FP	ACACTCGCAACTCTTACCCT	
	RP	CTTCTAGTTCGGACGCGGTG	

Table S5. Expression levels of *MYC*, *NFE2L2*, *G6PD*, and *TKT* genes in normal versus cancer tissue obtained from the Oncomine microarray Head-Neck data sets

Gene	Expression fold change			
	Estilo Head-Neck	Talbot Lung	TCGA Head-Neck	Ye Head-Neck
	Tongue SCC (31) versus Normal (26)	Tongue SCC (31) versus Normal (26)	HNSCC (385) versus Normal (74)	Tongue SCC (26) versus Normal (12)
NFE2L2	1.64**	1.51**	1.047***	1.103*
MYC	1.95**	1.70***	1.235***	1.613*
G6PD	1.74***	NS	1.045***	1.155*
TKT	1.88***	1.60***	NS	NS

NS, not statistically significant (t-test, p>0.05); SCC, squamous cell carcinoma.
Statistically significant (t-test, *p<0.05, **p<0.01, ***p<0.001)

Table S6. Nuclear to cytoplasmic NRF2 ratios in HNSCC patients with cigarette smoking, alcohol consumption and habitual chewing areca nut

Risk factor	Group	Carcinoma IRS ratio (Nuclear/Cytoplasm)			*p*-value
		N/C <1 (n=3)	N/C=1 (n=11)	N/C>1 (n=53)	
Cigarette smoking	No	0	6	8	0.0091
	Yes	3	5	45	
Alcohol consumption	No	1	6	10	0.0444
	Yes	2	5	43	
Betel quid chewing	No	0	6	8	0.0091
	Yes	3	5	45	

The nuclear/cytoplasmic ratio for NRF2 was calculated using the IRS score.

Table S7. Quantified relative metabolite abundance involved in redox regulation and nucleotide metabolism in NRF2-knockdown HNSCC cells.

Pathway	Metabolites	Ratio to the siControl
Redox Regulation	GSH	0.50
	GSSG	1.67
	$NADP^+$	0.77
	NADPH	0.64
Nucleotide Metabolism	CDP	1.01
	CMP	0.70
	CTP	0.88
	UDP	0.86
	UMP	0.74
	UTP	0.95
	AMP	0.84
	R5P	0.57

The results were normalized to total cell counts and the relative metabolite abundance was provided as a ratio to the siControl.

SUPPLEMENTARY REFERENCES

1. Leigh D, McBurney A, McIlwain H. Erythrocyte transketolase activity in the Wernicke-Korsakoff syndrome. Br J Psychiatry 139, 153-156 (1981).
2. Liu H, Huang D, McArthur DL, Boros LG, Nissen N, Heaney AP. Fructose induces transketolase flux to promote pancreatic cancer growth. Cancer Res 70, 6368-6376 (2010).
3. Meshalkina LE, Drutsa VL, Koroleva ON, Solovjeva ON, Kochetov GA. Is transketolase-like protein, TKTL1, transketolase? Biochim Biophys Acta 1832, 387-390 (2013).
4. Solovjeva ON, Kovina MV, Kochetov GA. Substrate inhibition of transketolase. Biochim Biophys Acta 1864, 280-282 (2016).
5. Dickson MA, Hahn WC, Ino Y, Ronfard V, Wu JY, Weinberg RA, Louis DN, Li PF, Rheinwald JG. Human keratinocytes that express hTERT and also bypass a p16(INK4a)-enforced mechanism that limits life span become immortal yet retain normal growth and differentiation characteristics. Mol Cell Biol. 20, 1436-47 (2000).
